# *Snail*-induced epithelial-to-mesenchymal transition of *MCF-7* breast cancer cells: systems analysis of molecular changes and their effect on radiation and drug sensitivity

**DOI:** 10.1186/s12885-016-2274-5

**Published:** 2016-03-18

**Authors:** Roman Mezencev, Lilya V. Matyunina, Neda Jabbari, John F. McDonald

**Affiliations:** Integrated Cancer Research Center, School of Biology, and Parker H. Petit Institute of Bioengineering and Biosciences, Georgia Institute of Technology, 315 Ferst Dr., Atlanta, GA 30332 USA

**Keywords:** Epithelial-to-mesenchymal transition, Snail, Slug, NF-κB, Drug resistance, Radiation sensitivity, MCF-7, Triple-negative breast-cancer, Reactive oxygen species, Glutathione

## Abstract

**Background:**

Epithelial-to-mesenchymal transition (EMT) has been associated with the acquisition of metastatic potential and the resistance of cancer cells to therapeutic treatments. *MCF-7* breast cancer cells engineered to constitutively express the zinc-finger transcriptional repressor gene *Snail* (*MCF-7-Snail* cells) have been previously shown to display morphological and molecular changes characteristic of EMT. We report here the results of a comprehensive systems level molecular analysis of changes in global patterns of gene expression and levels of glutathione and reactive oxygen species (ROS) in *MCF-7-Snail* cells and the consequence of these changes on the sensitivity of cells to radiation treatment and therapeutic drugs.

**Methods:**

*Snail*-induced changes in global patterns of gene expression were identified by microarray profiling using the Affymetrix platform (U133 Plus 2.0). The resulting data were processed and analyzed by a variety of system level analytical methods. Levels of ROS and glutathione (GSH) were determined by fluorescent and luminescence assays, and nuclear levels of NF-κB protein were determined by an ELISA based method. The sensitivity of cells to ionizing radiation and anticancer drugs was determined using a resazurin-based cell cytotoxicity assay.

**Results:**

Constitutive ectopic expression of *Snail* in epithelial-like, luminal A-type *MCF-7* cells induced significant changes in the expression of >7600 genes including gene and miRNA regulators of EMT. Mesenchymal-like *MCF-7-Snail* cells acquired molecular profiles characteristic of triple-negative, claudin-low breast cancer cells, and displayed increased sensitivity to radiation treatment, and increased, decreased or no change in sensitivity to a variety of anticancer drugs. Elevated ROS levels in *MCF-7-Snail cells* were unexpectedly not positively correlated with NF-κB activity.

**Conclusions:**

Ectopic expression of *Snail* in *MCF-7* cells resulted in morphological and molecular changes previously associated with EMT. The results underscore the complexity and cell-type dependent nature of the EMT process and indicate that EMT is not necessarily predictive of decreased resistance to radiation and drug-based therapies.

**Electronic supplementary material:**

The online version of this article (doi:10.1186/s12885-016-2274-5) contains supplementary material, which is available to authorized users.

## Background

Breast cancer is the most common female malignancy worldwide with an estimated 1.67 million new cases in 2012 [1]. Despite significant recent progress in the diagnosis and treatment of this biologically and clinically heterogeneous disease, breast cancer remains the most frequent cause of cancer death among women in less developed regions of the world and the second-leading cause of cancer death among women in developed nations [1, 2]. As is the case with most cancers, breast cancer-related deaths are primarily due to metastasis. Metastatic breast cancer (MBC) is present in ~6 % of patients at the time of initial diagnosis and eventually develops in 20–50 % of all breast cancer patients [2]. Since MBC is currently an incurable condition with median survival time of only 0.5–2.2 years, depending on subtype [3], it continues to be a challenging problem in both basic and clinical cancer research.

Epithelial-to-mesenchymal transition (EMT) is an essential process in normal embryonic development [4, 5] and has been associated with the acquisition of metastatic potential [6, 7] and the resistance of breast and other types of cancers to ionizing radiation [8] and anticancer drugs (reviewed in [9]). One of the genes frequently associated with EMT is the zinc-finger transcriptional repressor *Snail* (SNAI1) [10]. *Snail*, together with *Slug* (SNAI2) and *Smuc* (SNAI3), comprises the *Snail* family of transcription factors [11]. Previous studies indicate that both *Snail* and *Slug* may contribute to the progression of breast and other types of cancer by the down regulation of *E-cadherin* (CDH1) and other genes associated with the epithelial phenotype and the up regulation of genes associated with the mesenchymal phenotype (reviewed in [10, 12]).

In this study, we were interested in characterizing, on a molecular systems level, the role of *Snail* in breast cancer EMT and the consequence of this transition on the sensitivity of breast cancer cells to a variety of therapeutic treatments. Toward this end, we performed system level analyses of differences in global patterns of gene expression and therapeutic response profiles between two cell lines derived from the well-studied epithelial breast cancer cell line *MCF-7* (*M*ichigan *C*ancer *F*oundation-7) [13]. *MCF-7-Snail* is a derivative of *MCF-7* that has been stably transfected with a variant (*Snail-6SA*) of *Snail* and displays a mesenchymal-like morphology. *Snail-6SA* is a more stable protein than *wild-type Snail* and it has been shown to display constitutive activity and ability to induce EMT [14, 15]. *MCF-7-Control* is a derivative of *MCF-7* that has been transfected with an empty vector and displays the same epithelial morphology as the parental *MCF-7* cell line [14].

We report here that *MCF-7-Snail* cells display significant changes in the expression of several master regulators of EMT, including various zinc-finger and basic helix-loop-helix transcription factors, as well as members of the miR-200 family of microRNAs. While *MCF-7-Control* cells display molecular profiles characteristic of the luminal A (ER-positive, PR-positive, HER2-negative) breast cancer subtype, *MCF-7-Snail* cells were found to display molecular profiles characteristic of the aggressive triple-negative (ER-negative, PR-negative, HER2-negative), claudin-low breast cancer subtype. In addition, we found that relative to the *MCF-7-Control*, *MCF-7-Snail* cells display a higher level of cellular ROS, lower levels of GSH and NF-κB (nuclear factor *kappa*-light-chain-enhancer of activated *B* cells) activity, increased sensitivity to ionizing radiation and increased, decreased or no change in sensitivity to several anti-cancer drugs. Our results underscore the complexity of the EMT process in breast cancer cells and its consequence on cancer therapies.

## Methods

### Cell lines

*MCF-7-Snail* and *MCF-7-Control* cells, developed as previously described [14], were kindly provided by Dr. Valerie Odero-Marah (Clark Atlanta University). Transfected *MCF-7-Snail* and *MCF-7-Control* cells were selected from several clones to display the highest expression of Snail or the highest phenotypic similarity (doubling time) to the parental MCF-7 cells, respectively. Over-expression of Snail in *MCF-7-Snail* cells has been demonstrated using the western blot analysis [16]. Cells were routinely maintained in RPMI 1640 medium supplemented with 10 % FBS (Atlanta Biologicals, Lawrenceville, GA), 1 % antibiotic-antimycotic solution (Mediatech-Cellgro, Manassas, VA) and 400 μg/mL G418 (Geneticin, GIBCO) at 37 °C in a humidified atmosphere with 5 % CO_2_ and sub-cultured when they reach ~80 % confluence. In all experiments, cells were no more than four passages from the originally received *MCF-7-Snail* and *MCF-7-Control* cells.

### Expression analysis by microarray

*MCF-7-Snail* and *MCF-7-Control* cells (three replicates per cell line) were grown in the above-described medium and processed for microarray analysis using the Human Genome U133 Plus 2.0 Array (Affymetrix, Santa Clara, CA, USA). The resulting data were acquired as CEL files and processed with Expression Console software Build 1.2.1.20 (Affymetrix, Santa Clara, CA, USA) using the Affymetrix default analysis setting for PLIER and MAS 5.0 algorithms with annotation file HG-U133 Plus_2, Release 34 from 10/24/2013 (www.affymetrix.com). A detailed description of the microarray experiment and the resulting data are available in the Gene Expression Omnibus repository (GEO, http://www.ncbi.nlm.nih.gov/geo/) under the accession number GSE58252.

#### Differential expression analysis

Expression signals were converted to PLIER+16 and log_2_-transformed. Probe sets that displayed absent detection calls (MAS5.0 algorithm) across all chips were removed and log2 PLIER+16 values were used to identify genes differentially expressed between *MCF-7-Snail* and *MCF-7-Control* cells using the Significance Analysis of Microarrays (SAM) version 4.01 [17]. Genes were reported as differentially expressed at FDR = 2.12 % and absolute fold change (FC) ≥1.5. Differential gene expression was interpreted in the context of EMT and resistance to anticancer drugs using manually curated lists of 71 genes relevant to EMT and 53 genes relevant to anticancer drug resistance (these genes and their Affymetrix probe set IDs are listed in Additional file 1). The threshold for the expression signal intensities that allows identification of genes as highly likely “not expressed” was calculated by the “funnel-shaped procedure” described by Saviozzi et al. [18] and used to support lack of expression of selected genes (Additional file 2: Figure S1).

#### Pathway enrichment analysis

Probesets corresponding to differentially expressed genes were employed for enrichment analysis using the MetaCore suite 6.18 build 65,505 (Thomson Reuters, New York, NY, USA). Briefly, significantly perturbed pathways and process networks were identified by mapping differentially expressed genes onto manually curated GeneGO canonical pathway maps and cell process network models [19].

#### Interactome analysis

For each protein from the list of differentially expressed genes between *MCF-7-Snail* and *MCF-7-Control* cells, one step interaction neighbors from the global human interactome were identified using the MetaCore “interactome by protein function” tool (MetaCore suite 6.18 build 65,505; Thomson Reuters) and the local interactome was built by adding them to the protein interaction network built from genes differentially expressed between *MCF-7-Snail* and *MCF-7-Control* cells. Observed connectivity of each protein (network object) from this local interactome was compared to its expected connectivity based on the global human interactome and relative connectivity (connectivity ratio) was calculated to identify over-connected or under-connected network objects. Statistical significance of differences between observed and expected connectivities was evaluated using the hypergeometric test and multiplicity was controlled by the FDR procedure [20]. The list of over-connected network objects at FDR = 0.01 was reported.

#### Transcriptional network building

To elucidate complex relationships among the regulators of EMT in our dataset, a custom transcriptional network was built using the results of the differential expression analysis, previously reported associations between genes and EMT, as well as previously reported information on transcriptional regulation and influence on expression between selected network objects. Differentially expressed (i) transcription factors that were previously reported as major regulators of EMT, (ii) microRNA-200 family members, and (iii) epithelial or mesenchymal phenotype-associated genes coding for adherence junctions, tight junctions and intermediate filaments were employed to build the transcriptional network using the knowledge-based system MapEditor (MetaCore suite 6.18 build 65,505; Thomson Reuters). Relative expression data for network objects were color coded (red: up-regulation; blue: down-regulation in *MCF-7-Snail* relative to *MCF-7-Control* cells) and mapped on the transcriptional network. Network objects (genes) were connected in the network if their transcription regulation relationships were previously documented and included in the MetaCore knowledge base.

#### Gene Set Enrichment Analysis (GSEA)

To identify gene sets significantly enriched in a given phenotype (*MCF-7-Snail* or *MCF-7-Control*), GSEA [21] was performed on the data processed by PLIER+16 without any pre-filtering of probe sets, using categorical phenotype labels, gene set permutation type and signal-to-noise metrics. The following gene sets were employed in the analysis: C2: Curated Gene Sets (4722 gene sets) and C6: Oncogenic Signatures (189 gene sets) from the Molecular Signatures Database (http://www.broadinstitute.org/gsea/msigdb/collections.jsp).

In all enrichment analyses, the statistical significance of enrichment was evaluated using *p*-values calculated based on hypergeometric distribution and corrected for multiplicity using the false discovery rate (FDR) procedure. Unless stated otherwise, pathways, process networks or gene sets were considered to be significantly enriched, if their q-values were ≤ FDR threshold, for which the expected number of false positive entities was ≤1.

### MicroRNA expression analysis by qPCR

Relative expression of miRNA-429, miR-200a, miR-200b and miR-141 in *MCF-7-Snail* vs *MCF-7-Control* cells was determined by qPCR using specific TaqMan miRNA assays for miRNA-429, miR-200a, miR-200b and miR-141, and non-coding small nuclear RNA RNU6B as an endogenous reference (Applied Biosystems/Life Technologies, Carlsbad, CA). Total cell RNA was isolated using the mirVana miRNA Isolation Kit (Ambion, Foster City, CA, USA) and cDNAs were prepared using the miRNA-specific stem-loop RT primers and TaqMan MicroRNA Reverse Transcription Kit following the manufacturer’s recommendation. Thereafter, cDNA was amplified using the TaqMan Universal Master Mix II with UNG in the CFX96 Real Time PCR Detection System (BioRad, Hercules, CA) following the manufacturer’s recommendation. Expressions of individual miRNAs in *MCF-7-Snail* relative to *MCF-7-Control* cells was calculated from the threshold cycles using the REST 2009 Software (Qiagen, Valencia, CA, USA) [22] and expressed as means, and the 95 % confidence intervals calculated by bootstrapping technique without normality or symmetrical distribution assumptions. *P*-values determined by a randomization test represent the probability that the observed difference in expression between *MCF-7-Snail* and *MCF-7-Control* cells is due to chance.

### Determination of radiation sensitivity

One hundred thousand cells were plated in 2.5 mL of RPMI 1640 medium supplemented with 10 % FBS in 35 mm tissue culture dishes (Corning Incorporated, Corning, NY, USA). After 24 h, the cultures were irradiated in an RS-2000 X-ray irradiator (Rad Source Technologies, Suwanee, GA) at 160 kV and 25 mA on an aluminum specimen shelf four at dose rate ~ 311 cGy/min and single dose levels 2 Gy (39 s), 4 Gy (77 s) and 8 Gy (154 s). Control medium was irradiated at 4 Gy. After the irradiation, cells were allowed to grow for 72 h at 37 °C in a humidified atmosphere with 5 % CO_2_. For quantification of viable cells, 200 μL of Tox-8 reagent were added to each dish and incubated for 2.5 h at 37 °C in a humidified atmosphere with 5 % CO_2_. Thereafter, the specimens were transferred to a 96-well plate (200 μL/well) and viable cells were quantified via fluorescence at 560 nm excitation and 590 nm emission. The results were expressed as % of non-irradiated control.

### Determination of cell cycle distribution

Cells plated in parallel with cells used in the radiation sensitivity experiment were cultured for 24 h, harvested by trypsinization, fixed and stained for DNA analysis by flow cytometry as previously described [23]. Cell cycle distribution was determined by deconvolution of DNA content histograms, after discrimination of doublets and other cellular aggregates by FlowJo 7.6.5 software (Tree Star, Inc., Ashland, OR, USA) using the Dean-Jet-Fox Model. For each cell line, the flow cytometry DNA analysis was performed on three independent cell cultures and the results are presented as means from these three experiments.

### Determination of intracellular level of ROS

*MCF-7-Snail* and *MCF-7-Control* cells in the RPMI-1640 medium supplemented with 10 % FBS (20,000 cells/mL) were plated into 96-well black-walled plate (100 μL/well) and incubated for 48 h at 37 °C in a humidified atmosphere with 5 % CO_2_. Thereafter, the medium was removed and 10 μM solution of 2′,7′-dichlorodihydrofluorescein diacetate (H2DCF-DA, Molecular Probes, Inc., Eugene, OR) in PBS was added to each well (100 μL/well). H2DCF-DA is a general oxidative stress indicator that can detect several types of ROS including hydrogen peroxide, hydroxyl radicals and peroxynitrite [24]. Cells were incubated for additional 30 min at 37 °C in a humidified atmosphere with 5 % CO2 and the fluorescence of the ROS-sensitive dye was measured by a Synergy 4 microplate reader (Biotek, Winooski, VT) with filter set 485/20 nm (excitation), 528/20 nm (emission) and 510 nm full-size mirror. Fluorescence intensity corresponding to the ROS signal was normalized to the quantity of viable cells per well as determined by TOX-8 assay and expressed as mean±SD.

### Determination of cellular glutathione

*MCF-7-Snail* and *MCF-7-Control* cells in DMEM medium (glutathione-free) supplemented with 10 % FBS (20,000 cells/mL) were plated onto the tissue culture-treated 96-well white-walled plate (100 μL/well) and incubated for 48 h at 37 °C in a humidified atmosphere with 5 % CO_2_.

Reduced glutathione (GSH) and total cellular glutathione (GSH+GSSG) in *MCF-7-Snail* and *MCF-7-Control* cells were quantified using the GSH-Glo Glutathione Assay (Promega, Madison, WI, USA). In this assay, the luciferin derivative Luc-NT is converted in the presence of GSH and glutathione S-transferase (GST) to luciferin that generates a luminescent signal in a coupled reaction catalyzed by firefly luciferase. The assay was performed following the manufacturer’s instructions for adherent cell cultures.

Total cellular glutathione was determined after reduction of GSSG to GSH with tris(2-carboxyethyl) phosphine (TCEP, final concentration 1 mM). The luminescence signal after subtraction of blanks (net RLU) was normalized to the number of viable cells determined by resazurin-based cell viability assay TOX-8 (Sigma–Aldrich, St. Louis, MO). All experiments were performed in triplicate.

### Determination of the level of nuclear NF-κB

*MCF-7-Snail* and *MCF-7-Control* cells each in three replicated cultures were grown in full growth medium to ~80 % confluence, harvested by scraping and processed to obtain nuclear protein extracts using the CelLytic NuCLEAR Extraction Kit (Sigma-Aldrich) following the manufacturer’s protocol. Protein concentration in nuclear extracts was determined using the Pierce 660 nm Protein Assay (Thermo Scientific, Rockford, IL). NF-κB (p50 subunit) was determined in nuclear protein extracts by an ELISA-based assay using the NF-κB (human p50) Transcription Factor Assay Kit (Cayman Chemical Company, Ann Arbor, MI, USA) in 96-well assay format following the manufacturer’s protocol. After developing plates, the stop solution was added and signals corresponding to the p50 protein levels were read as A_450_ - A_570_. Concentration of nuclear NF-κB was expressed as A_450_ - A_570_ corrected for non-specific binding signal and normalized to protein concentration in nuclear extracts. The results were expressed as means±SDs.

### Determination of drug sensitivity

Sensitivity of *MCF-7-Snail* and *MCF-7-Control* cells to the cytotoxic effects of selected conventional anticancer drugs was evaluated using the resazurin-based in vitro toxicology assay kit TOX-8 (Sigma–Aldrich) as previously described [23]. Aliquots of cell suspensions (100 μL/well) were plated onto 96-well black-walled plates at 30,000 cells/mL in RPMI 1640 medium supplemented with 10 % FBS, 1 % antibiotic-antimycotic solution and 400 μg/mL G418. Tested compounds were diluted from the following stock solutions: vincristine (VCR) - 0.4 mM in DMSO; doxorubicin (DOX) – 2 mM in DMSO; methotrexate (MTX) – 1 mM in DMSO; gemcitabine (GEM) – 10 mM in H_2_O; mitomycin C (MMC) – 10 mM in DMSO; 5-fluorouracil (5-FU) – 16.5 mM in H_2_O; cisplatin (CPT) – 1.7 mM in 0.9 % NaCl/H_2_O. Tested compounds dissolved in growth medium at a concentration twice the desired final concentration were added in quadruplicates at 100 μL volumes per well. Incubation of cells with drugs or control medium proceeded for 72 h. After that, 20 μL of the TOX-8 reagent were added to each well and incubated for the next 3 h. The increase of fluorescence was measured at a wavelength of 590 nm using an excitation wavelength of 560 nm. The emission of control wells (no drug treatment) after the subtraction of a blank was taken as 100 % and the results for treatments were expressed as a percentage of the control. The experiment was performed four times and GI50 values (concentrations of tested agents that inhibited growth of cell cultures after 72-h incubation to 50 % of the untreated control) were determined by non-linear regression of log-transformed data using a normalized response-variable slope model (GraphPad Prism 5.01; GraphPad Software, Inc.) and expressed as mean ± SD.

### Statistical analyses

Unless stated otherwise, the statistical significance of differences between means of continuous data was evaluated by Welch-corrected t-test and considered significant for two-tailed *p*-values <0.05. In the analysis of microarray data, multiplicity of statistical tests was corrected by the FDR approach and the discoveries were considered significant if their rank and q-value would allow not more than one false discovery. When multiple comparisons were performed in other experiments, *p*-values were adjusted by the Šidák-Holm approach and considered significant for *p*_adj_ <0.05. Unless stated otherwise, results of continuous variables were expressed as means±SD.

## Results

### *MCF-7-Snail* cells display morphological and molecular changes characteristic of EMT

**Fig. 1:**
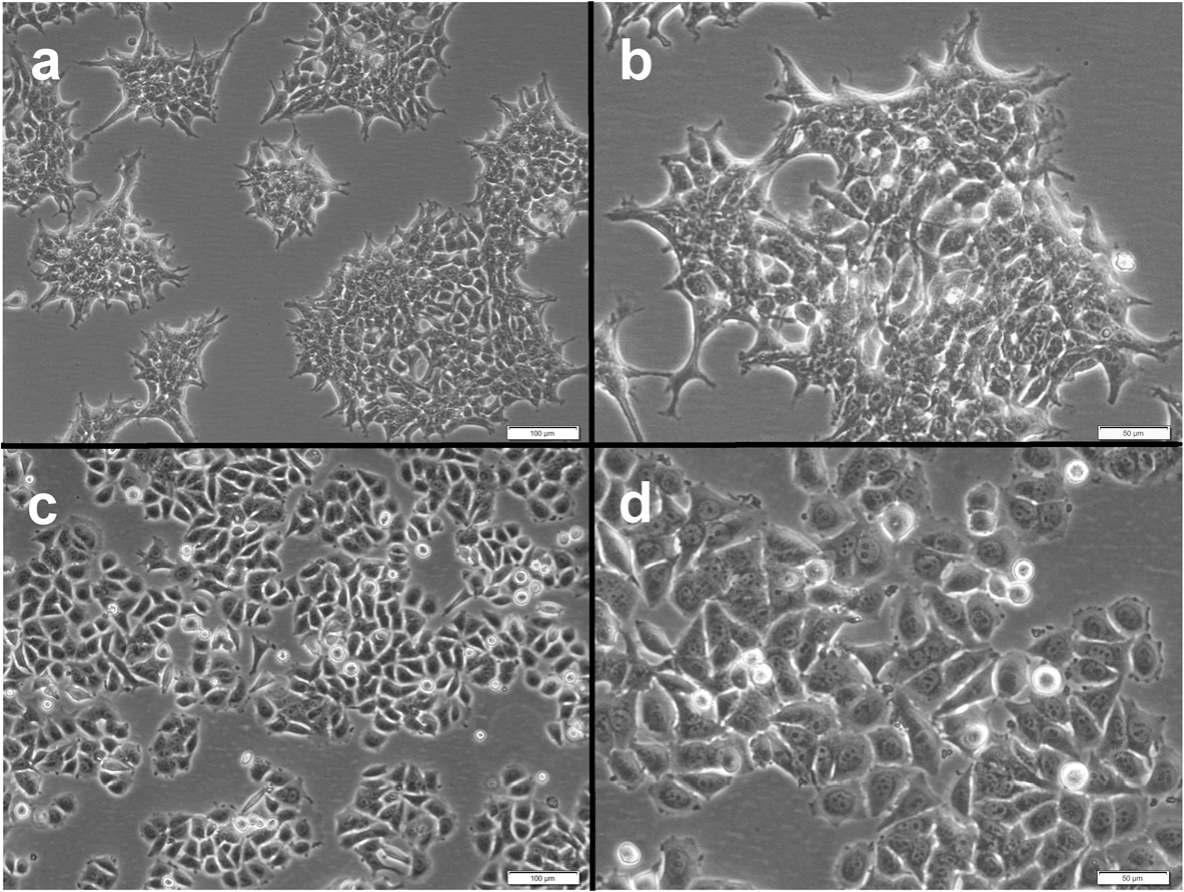
Brightfield (phase contrast) micrographs of *MCF-7-Snail* and *MCF-7-Control* cells. Shown are *MCF-7-Snail* (**a, b**) and *MCF-7 Control* cells (**c, d**). Magnification: 100× (**a, c**) or 200× (**b, d**). Scale bars: 100 μm, (**a, c**) and 50 μm (**b, d**)

*MCF-7-Snail* cells display an elongated morphology characteristic of mesenchymal-like breast cancer cells (Fig. 1a-b). In contrast *MCF-7-Control* cells, like their parental *MCF-7* cells, display the classic “cobble-stone” morphology characteristic of breast cancer epithelial cells (Fig. 1c-d). Consistent with these morphological differences, *MCF-7-Snail* and *MCF-7-Control* cells are remarkably different in their respective patterns of gene expression (Additional file 2: Figure S2).

**Fig. 2:**
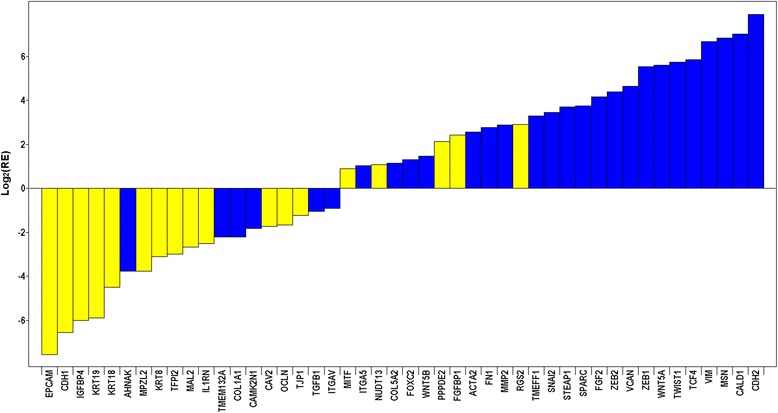
Relative expression of a subset of the 71 EMT-related genes in *MCF-7-Snail* vs *MCF-7-Control* cells. Results shown in log2 scale (from microarray data). Color-coding: *Yellow* = epithelial phenotype-associated genes; *blue* = mesenchymal phenotype-associated genes

Among 38,226 probe sets included in the differential expression analysis, over 12,000 probe sets corresponding to 7602 genes were found to display statistically significant differences in expression (4242 up-regulated; 3291 down-regulated; 69 discordant) between *MCF-7-Snail* and *MCF-7-Control* cells (FDR = 2.12 % and |FC| ≥1.5 (Additional file 2: Figure S3, Additional files 3 and 4). Among the genes significantly differentially expressed are many that have been previously implicated in EMT, including transcription factors Slug, Zeb1, Zeb2, Twist1 [25] and TCF4 [26] (Fig. 2). Among the differentially expressed genes 69 genes displayed discordant expression with some probe sets detecting their up regulation and other probe sets detecting down regulation (Additional file 4). The over-expression of SNAI1 (Snail) and SNAI2 (Slug) genes in *MCF-7-Snail* relative to *MCF-7-Control* cells was confirmed by qPCR (Additional file 2: Figure S4 and Additional file 2: Method S1. We have previously reported the up-regulation of mesenchymal markers CDH2 and VIM and down-regulation of epithelial marker CDH1 in *MCF-7-Snail* cells by RT-PCR [27].

### The miR-200 family of microRNAs are significantly down-regulated in *MCF-7-Snail* cells

The regulatory role of microRNAs in EMT is an area of growing interest for both developmental and cancer biologists [28]. Members of the miR-200 family of microRNAs are of particular interest because they have been previously shown to target genes that play central roles in EMT (e.g.*, Zeb1, Zeb2, Slug*) [29, 30]. In addition, more recent studies have demonstrated that miR-429 and other members of the miR-200 family are down regulated in ovarian cancer mesenchymal-like cells and that ectopic over-expression of these miRNAs in these cells is sufficient to induce mesenchymal-to-epithelial transition (MET) [31–33]. In light of these findings and because *Snail*-induced repression of miR-200 family miRNAs has recently been implicated with EMT in embryonic stem cells (ESC) [34], we examined levels of miR-200 members in *MCF-7-Snail* cells relative to controls.

**Fig. 3:**
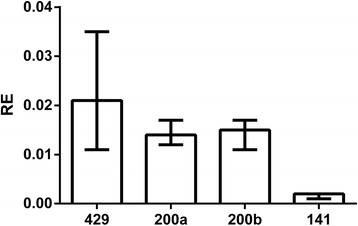
Relative expression of miRNA 200 family members in *MCF-7-Snail* vs *MCF-7 Control* cells. Relative expression (RE) determined by qPCR. Error bars: 95 % CI (*N* = 4 replicates). *P*-values from randomization test: miR-429 (*p* = 0.008), miR-200a (*p* = 0.016), miR-200b (0.022), miR-141 (*p* = 0.015)

**Fig. 4:**
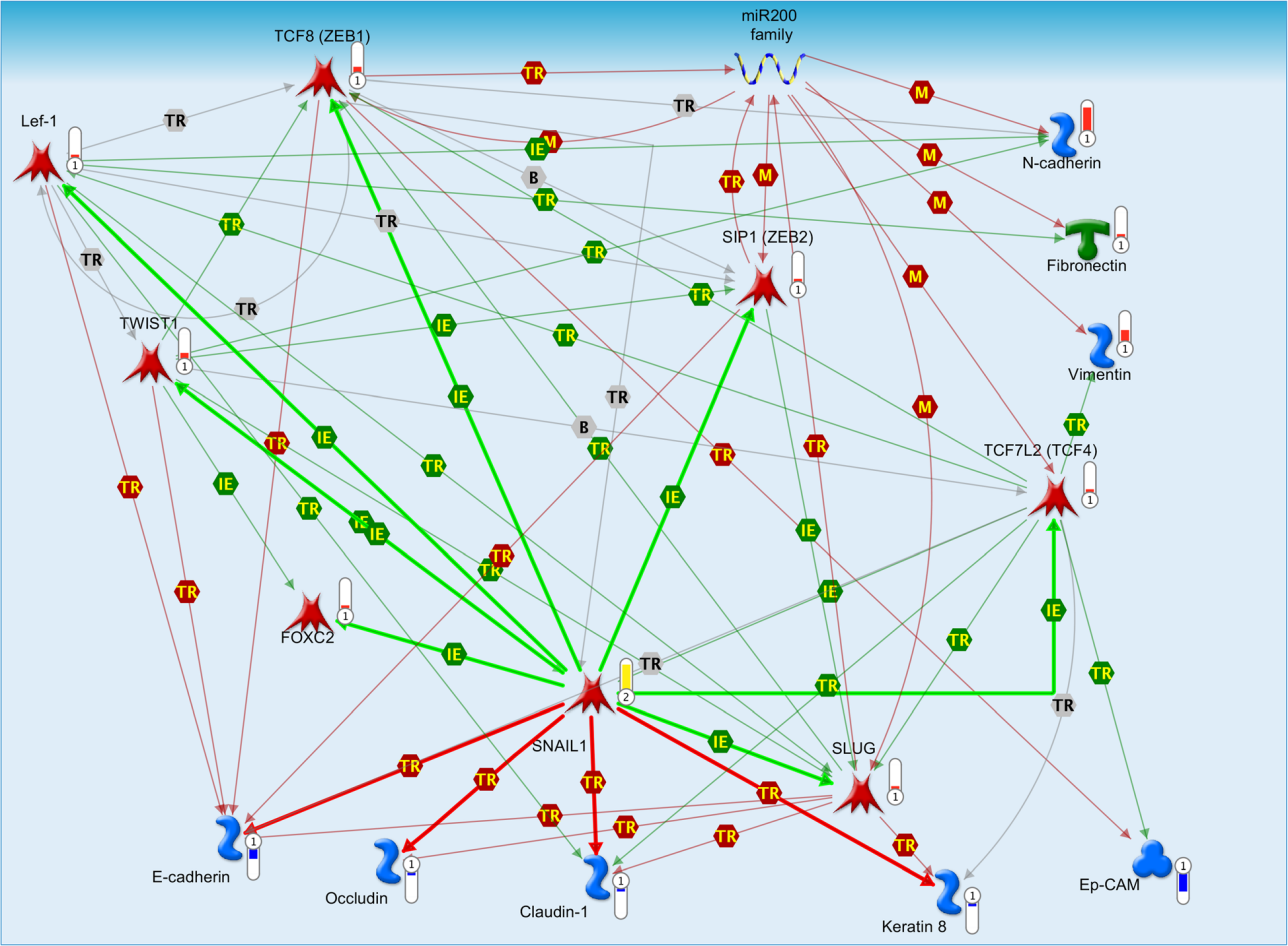
Complex regulatory interplay among transcription factors, miRNA 200 family members and E/M-phenotype related genes. Map created from genes differentially expressed between *MCF-7-Snail* and *MCF-7-Control* cells using MapEditor (Thomson Reuters, New York, NY, USA) to connect network objects based on previously reported associations. Legend for edges: *TR* transcriptional regulation, *IE* influence on expression, *M* microRNA binding. Green edge – activation; red edge repression. Edges originating or ending at SNAI1 are depicted as thick lines. Thermometers: *red* = network object up-regulated in *MCF-7-*Snail cells; *blue* = network object down-regulated in *MCF-7-Snail* cells; *yellow* = network object identified as over-connected to the list of differentially expressed genes. For more details on legend https://portal.genego.com/legends/MetaCoreQuickReferenceGuide.pdf

The results of comparative qPCR expression analyses of four miRNA-200 family members in *MCF-7-Snail* and *MCF-7-Control* cells indicate that levels of miR-200 family microRNAs are consistently and significantly reduced in the mesenchymal-like *MCF-7-Snail* cells (Fig. 3). Since a number of EMT associated genes are known or predicted targets of miR-200 family members (Fig. 4), these findings suggest that down regulation of members of the miR-200 family of microRNAs may contribute to the regulatory changes associated with EMT in *MCF-7-Snail* cells.

### Systems analysis provides evidence of a complex regulatory interplay between EMT-associated genes and miR-200 family miRNAs in *MCF-7-Snail* cells

Transcriptional network analysis of genes and miR-200 miRNAs differentially expressed between *MCF-7-Snail* and *MCF-7-Control* cells suggests a complex regulatory relationship among EMT-associated transcription factors, miR-200 family members and various cytoskeletal and junction proteins previously associated with the epithalial/mesenchymal phenotype (Fig. 4).

**Fig. 5:**
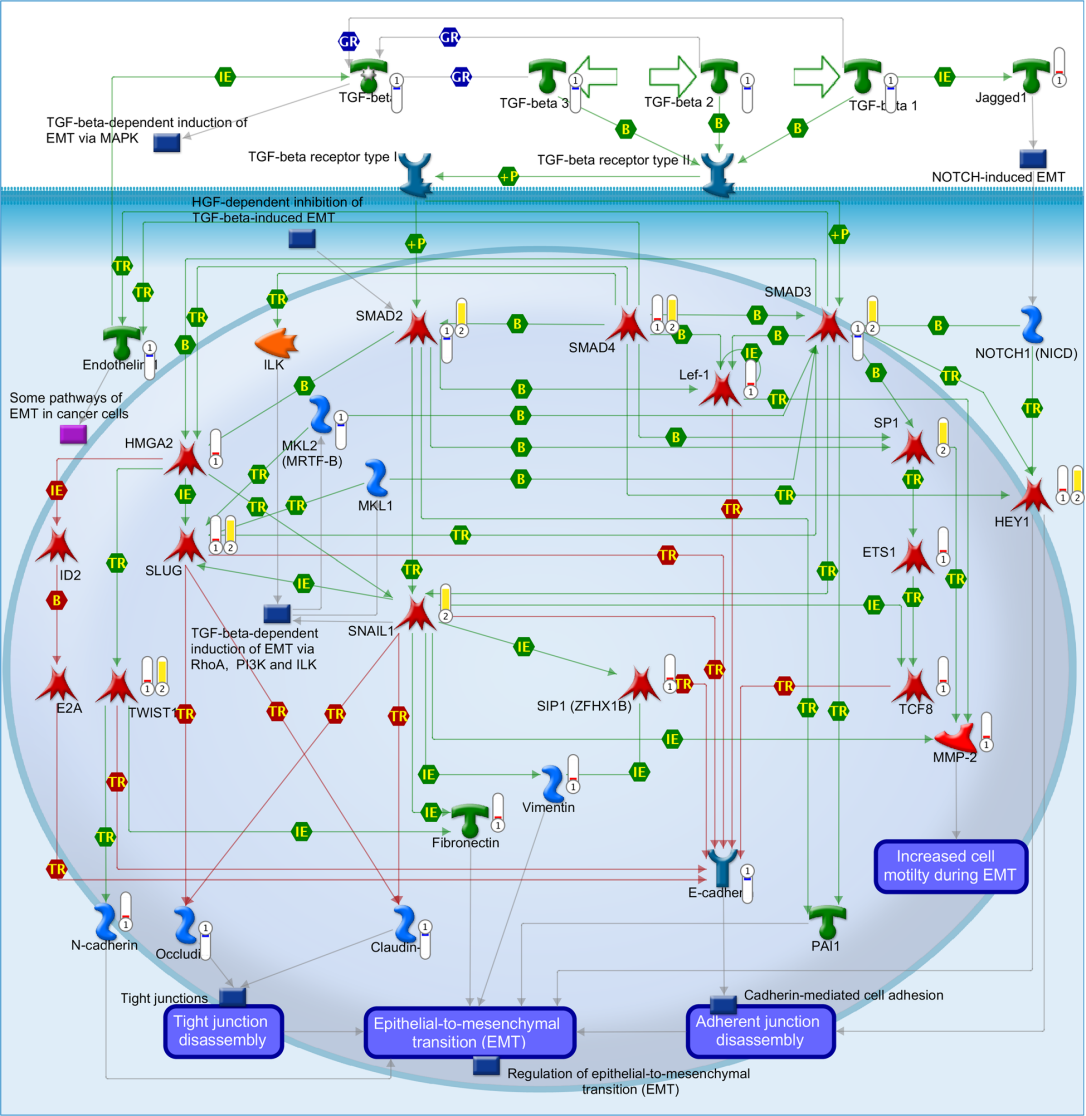
GeneGO pathway map “Development_TGF-beta-dependent induction of EMT via SMADs” is significantly enriched for genes differentially expressed between *MCF-7-Snail* and *MCF-7-Control* cells. Thermometers: *red* = object up-regulated in *MCF-7-*Snail cells; *blue* = object down-regulated in *MCF-7-Snail* cells; *yellow* = network object identified as over-connected to the list of differentially expressed genes. For more details on legend see https://portal.genego.com/legends/MetaCoreQuickReferenceGuide.pdf

Pathway Enrichment Analysis of the 7634 network objects that were recognized by MetaCore suite among 7602 differentially expressed genes between *MCF-7-Snail* and *MCF-Control* cells, identified 18 significantly enriched pathways for both up- and down-regulated genes (Table 1, FDR = 0.06089). Likewise, six significantly enriched pathways were identified for up-regulated genes only (Additional file 2: Table S1, FDR = 0.1677) and 69 pathways for down-regulated genes only (Additional file 2: Table S2, FDR = 0.01514). Mapping up- and down-regulated genes onto GeneGO Process Network Maps identified 30 significantly enriched networks (Table 2, FDR = 0.03104). As expected, the enriched pathways and networks include those related to EMT (e.g., Fig. 5, Tables 1 and 2). Additionally implicated were other related cellular processes including the Wnt-signaling, Hedgehog signaling, estrogen receptor-mediated signaling, NOTCH-signaling, ERBB-signaling, the endoplasmic reticulum stress pathway, and reactive oxygen species (ROS)-associated processes (Tables 1 and 2).

**Table 1:** GeneGO pathway maps significantly enriched for both up- and down-regulated genes in *MCF-7-Snail* vs *MCF-7-Control* cells (FDR = 0.06089); P/T – differentially expressed genes mapped to a given map/total number of genes in a map

#	Pathway map	*p*-value	FDR	P/T
1	Chemotaxis_C5a-induced chemotaxis	6.298E-05	3.136E-02	21/27
2	ENaC regulation in normal and CF airways	7.539E-05	3.136E-02	26/36
3	Development_TGF-beta-dependent induction of EMT via SMADs	1.394E-04	3.865E-02	25/35
4	Development_Thromboxane A2 signaling pathway	2.847E-04	5.199E-02	25/36
5	Muscle contraction_Relaxin signaling pathway	3.125E-04	5.199E-02	26/38
6	Ca(2+)-dependent NF-AT signaling in cardiac hypertrophy	5.485E-04	5.627E-02	25/37
7	Main growth factor signaling cascades in multiple myeloma cells	5.485E-04	5.627E-02	25/37
8	Cytoskeleton remodeling_RalA regulation pathway	5.733E-04	5.627E-02	19/26
9	Regulation of CFTR activity (normal and CF)	6.448E-04	5.627E-02	28/43
10	Signal transduction_Calcium signaling	8.247E-04	5.627E-02	22/32
11	Apoptosis and survival_HTR1A signaling	1.003E-03	5.627E-02	25/38
12	Development_A1 receptor signaling	1.003E-03	5.627E-02	25/38
13	Transcription_CREB pathway	1.045E-03	5.627E-02	26/40
14	Transport_Clathrin-coated vesicle cycle	1.054E-03	5.627E-02	40/68
15	PTMs in IL-17-induced CIKS-independent signaling pathways	1.079E-03	5.627E-02	27/42
16	Signal transduction_Activation of PKC via G-Protein coupled receptor	1.107E-03	5.627E-02	28/44
17	Apoptosis and survival_Endoplasmic reticulum stress response pathway	1.150E-03	5.627E-02	31/50
18	Development_Hedgehog and PTH signaling pathways in bone and cartilage development	1.345E-03	6.089E-02	20/29

**Table 2:** GeneGO Process Networks significantly enriched for both up- and down-regulated genes in *MCF-7-Snail* vs *MCF-7-Control* cells (FDR = 0.03104). P/T – differentially expressed genes mapped to a given map/total number of genes in a map

#	Network	*p*-value	FDR	P/T
1	Signal transduction_WNT signaling	1.774E-07	2.622E-05	100/170
2	Cell adhesion_Cell junctions	3.278E-07	2.622E-05	89/149
3	Cytoskeleton_Regulation of cytoskeleton rearrangement	5.064E-07	2.701E-05	104/181
4	Cell adhesion_Cadherins	1.194E-06	4.777E-05	100/175
5	Cytoskeleton_Actin filaments	3.552E-06	1.137E-04	99/176
6	Development_Hedgehog signaling	1.016E-05	2.598E-04	129/244
7	Reproduction_FSH-beta signaling pathway	1.136E-05	2.598E-04	86/152
8	Development_Neurogenesis_Axonal guidance	4.544E-05	9.088E-04	115/219
9	Signal transduction_Androgen receptor nuclear signaling	5.687E-05	9.838E-04	70/123
10	Development_Regulation of angiogenesis	6.148E-05	9.838E-04	109/207
11	Cell adhesion_Attractive and repulsive receptors	1.134E-04	1.649E-03	91/170
12	Development_Neurogenesis_Synaptogenesis	6.650E-04	7.983E-03	92/179
13	Cell cycle_G1-S Growth factor regulation	6.820E-04	7.983E-03	96/188
14	Development_Ossification and bone remodeling	7.539E-04	7.983E-03	80/153
15	Signal transduction_ESR1-nuclear pathway	8.718E-04	7.983E-03	104/207
16	Development_EMT_Regulation of epithelial-to-mesenchymal transition	8.718E-04	7.983E-03	104/207
17	Signal Transduction_Cholecystokinin signaling	8.781E-04	7.983E-03	52/93
18	Signal transduction_ERBB-family signaling	8.981E-04	7.983E-03	40/68
19	Muscle contraction_Relaxin signaling	2.135E-03	1.798E-02	37/64
20	Apoptosis_Endoplasmic reticulum stress pathway	2.366E-03	1.819E-02	44/79
21	Development_Neurogenesis in general	2.387E-03	1.819E-02	93/187
22	Neurophysiological process_Circadian rhythm	2.520E-03	1.833E-02	30/50
23	Cardiac development_FGF_ErbB signaling	2.858E-03	1.988E-02	64/123
24	Cardiac development_Wnt_beta-catenin, Notch, VEGF, IP3 and integrin signaling	3.396E-03	2.181E-02	70/137
25	Signal transduction_ESR1-membrane pathway	3.425E-03	2.181E-02	43/78
26	Cardiac development_Role of NADPH oxidase and ROS	3.669E-03	2.181E-02	64/124
27	Reproduction_Gonadotropin regulation	3.680E-03	2.181E-02	81/162
28	Signal transduction_NOTCH signaling	4.606E-03	2.629E-02	109/227
29	Signal transduction_Androgen receptor signaling cross-talk	4.766E-03	2.629E-02	35/62
30	Reproduction_Male sex differentiation	5.820E-03	3.104E-02	111/233

To further explore genes differentially expressed between *MCF-7-Snail* and *MCF-7-Control* cells, we examined their paired (binary) protein interactions with those in the MetaCore human protein interaction network [20]. This interactome analysis identified 164 significantly over-connected, but no significantly under-connected human interactome proteins (Additional file 5). The list of over-connected proteins includes: (i) Snail, Slug and Twist1 consistent with their previously recognized role in EMT [25]; (ii) GSK3B, RYK, β-catenin (CTNNB1) and TCF7L2 (TCF4) consistent with a role in the Wnt signaling pathway in *Snail*-induced EMT [10]; (iii) ESR1, PGR (PR) and androgen receptor suggesting a role of the sex-hormone-receptor-mediated signaling pathway; (iv) cancer-associated transcription factors p53 (TP53) and c-Myc (MYC), and (iv) the pluripotency-associated transcription factors KLF-4, Oct3/4, Nanog, Sox2 [35, 36].

We next focused on transcription factor sub-networks represented among genes significantly differentially expressed between *MCF-7-Snail* and *MCF-7-Control* cells. This analysis identified 31 transcription factor-centered networks (Additional file 6) confirming a likely role of several transcription factors previously implicated in EMT (MYC, Oct3/4, ESR1, p53, Nanog, Sox2 and TCF-4), in addition to others (CREB1, SP1, NF-κB, and ETS1). For example, among the 294 genes differentially expressed between *MCF-7-Snail* and *MCF-7-Control* cells known to be transcriptionally activated by MYC, 240 (~82 %) were up regulated in *MCF-7-Snail* cells (Additional file 7). Of the 162 genes differentially expressed between *MCF-7-Snail* and *MCF-7-Control* cells known be transcriptionally repressed by MYC, 107 (~66 %) were found to be down regulated in *MCF-7-Snail* cells (Additional file 7). These results suggest a significant role and up-regulated activity of MYC in *Snail*-induced EMT in breast cancer.

### *MCF-7-Snail* cells display molecular profiles characteristic of the triple-negative breast cancer subtype

**Fig. 6:**
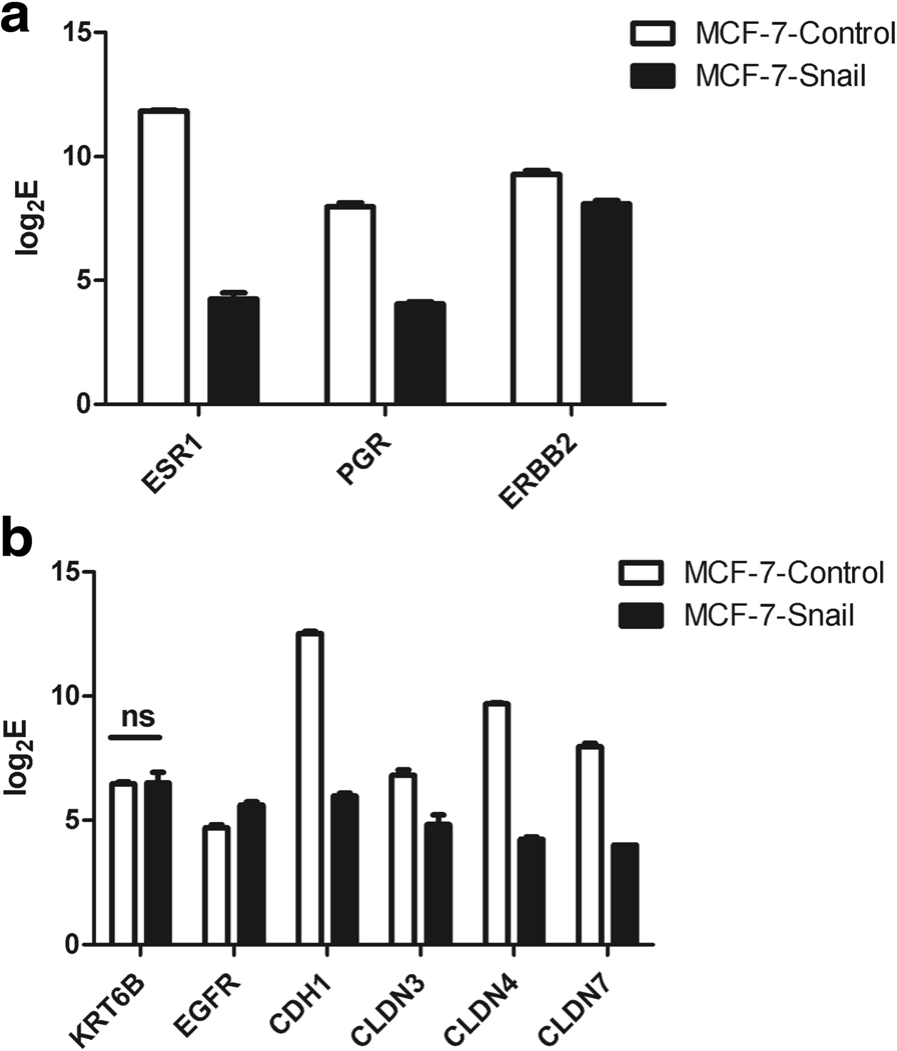
Expression of molecular markers for classification of breast cancer subtypes and for sub-typing of triple-negative breast cancers. Shown are log_2_E - logarithm of PLIER+16-processed expression signals of molecular markers for breast cancer subtypes (**a**) and for sub-typing of triple-negative breast cancers (**b**); *ns* difference between *MCF-7-Snail* and *MCF-7-Control* cells was not significant (all other markers are significantly differentially expressed between *MCF-7-Snail* and *MCF-7*-*Control* cells at FDR = 2.12 % and absolute fold change (FC) ≥1.5)

Estrogen receptor 1 (*ESR1*), progesterone receptor (*PGR*) and *ERBB2* genes were found to be significantly down-regulated in *MCF-7-Snail* relative to *MCF-Control* cells (Fig. 6a). In addition, the intensities of ESR1 and PGR transcripts were below computed threshold levels, indicating that these genes are not significantly expressed in *MCF-7-Snail* cells (Additional file 2: Figure S1B).

Further evidence of the involvement of ESR1 in the molecular changes underlying the phenotypic differences between *MCF-7-Snail* and *MCF-7-Control* cells, is the observation that *ESR1* is significantly over-connected with genes differentially expressed between the two cell lines (Additional file 5). In addition, of the 262 genes differentially expressed between *MCF-7-Snail* and *MCF-7-Control* cells known to be transcriptionally activated by ESR1, 172 (~66 %) were found to be down regulated in *MCF-7-Snail* cells (Additional file 8). Collectively, these findings consistently indicate that *ESR1*-mediated signaling is significantly reduced in *MCF-7-Snail* relative to *MCF-Control* cells and we have confirmed the down-regulation of ESR1 in *MCF-7-Snail* cells by qPCR (Additional file 2: Figure S5a and Additional file 2: Method S2).

Although the expression intensity of the *ERBB2* gene that encodes HER-2/neu protein appears to exceed the calculated expression threshold (Additional file 2: Figure S1B), we conclude that the *ERBB2* gene is not significantly expressed in *MCF-7-Snail* cells for the following two reasons: (i) the probe set that detected ERBB2 expression in our dataset (216836_s_at) is not specific and may also detect other transcripts, and (ii) differential expression analysis identified the *ERBB2* gene as down regulated upon ectopic expression of *Snail* in *MCF-7* cells, which are known to be HER-2/neu negative [37]. This conclusion is further supported by the quantification of total HER-2/neu protein in *MCF-7-Snail* and *MCF-7-Control* cells using the ELISA (Additional file 2: Figure S6 and Additional file 2: Method S3).

Taken together our results indicate that while *MCF-7*-*Control* cells display molecular profiles characteristic of the luminal A subtype of breast cancer (ER-positive, PR-positive and HER-2/neu-negative) [37, 38], *MCF-7-Snail* have acquired profiles characteristic of the triple-negative (ER-negative, PR-negative, HER-2/neu-negative), breast cancer subtype.

Gene Set Enrichment Analysis (GSEA) further supports this conclusion in that the enrichment of gene sets previously associated with the ductal-invasive, non-luminal, mesenchymal and/or metaplastic phenotype (e.g., activation of KRAS, LEF1, EGFR and suppression of PTEN functions) are also significantly enriched in *MCF-7-Snail* cells. Conversely, GSEA of *MCF-7-Control* cells demonstrated enrichment of gene sets previously associated with the estrogen receptor positive, luminal type, and epithelial differentiated phenotype (Additional file 9).

Triple-negative breast cancers have been sub-classified into specific molecular subtypes that include basal-like breast cancers (CK5/6-positive and/or EGFR-positive) [39] and claudin-low breast cancers exhibiting low expression of the *CDH1* and claudin 3, 4 and 7 genes [40]. Cytokeratins 5 and 6 were not found among genes differentially expressed between *MCF-7-Snail* and *MCF-7-Control* cells. Consistent with the previously reported absence of the expression of CK5/6 in *MCF-7* cells [41], our result suggests that these genes are not significantly expressed in either *MCF-7-Snail* or *MCF-7-Control* cells. Although the *EGFR* gene appears to be expressed over the calculated threshold, and up-regulated in MCF-7-Snail cells (Additional file 2: Figure S5b, Additional files 3 and 4), its signal intensity is low (Additional file 2: Figure S1B). Moreover, the *ITGB4* gene previously associated with the basal-like subtype [42] is down regulated in *MCF-7-Snail* cells (Additional files 3 and 4).

Overall, our gene expression results do not support the basal-like phenotype for *MCF-7-Snail* cells. In contrast, genes whose down regulation is known to be associated with the claudin-low subtype (*CLDN3, CLDN4, CLDN7, CDH1,* and *ITGB4*) [42] are all significantly down regulated in *MCF-7-Snail* cells (Fig. 6b, Additional file 2: Figure S1B, Additional file 3 and Additional file 4). Collectively, our findings indicate that ectopic expression of *Snail* has induced the transformation of *MCF-7* cells from the luminal A-like cells to the claudin-low triple-negative breast cancer subtype.

### Levels of reactive oxygen species (ROS) are significantly elevated in *MCF-7-Snail* cells

**Fig. 7:**
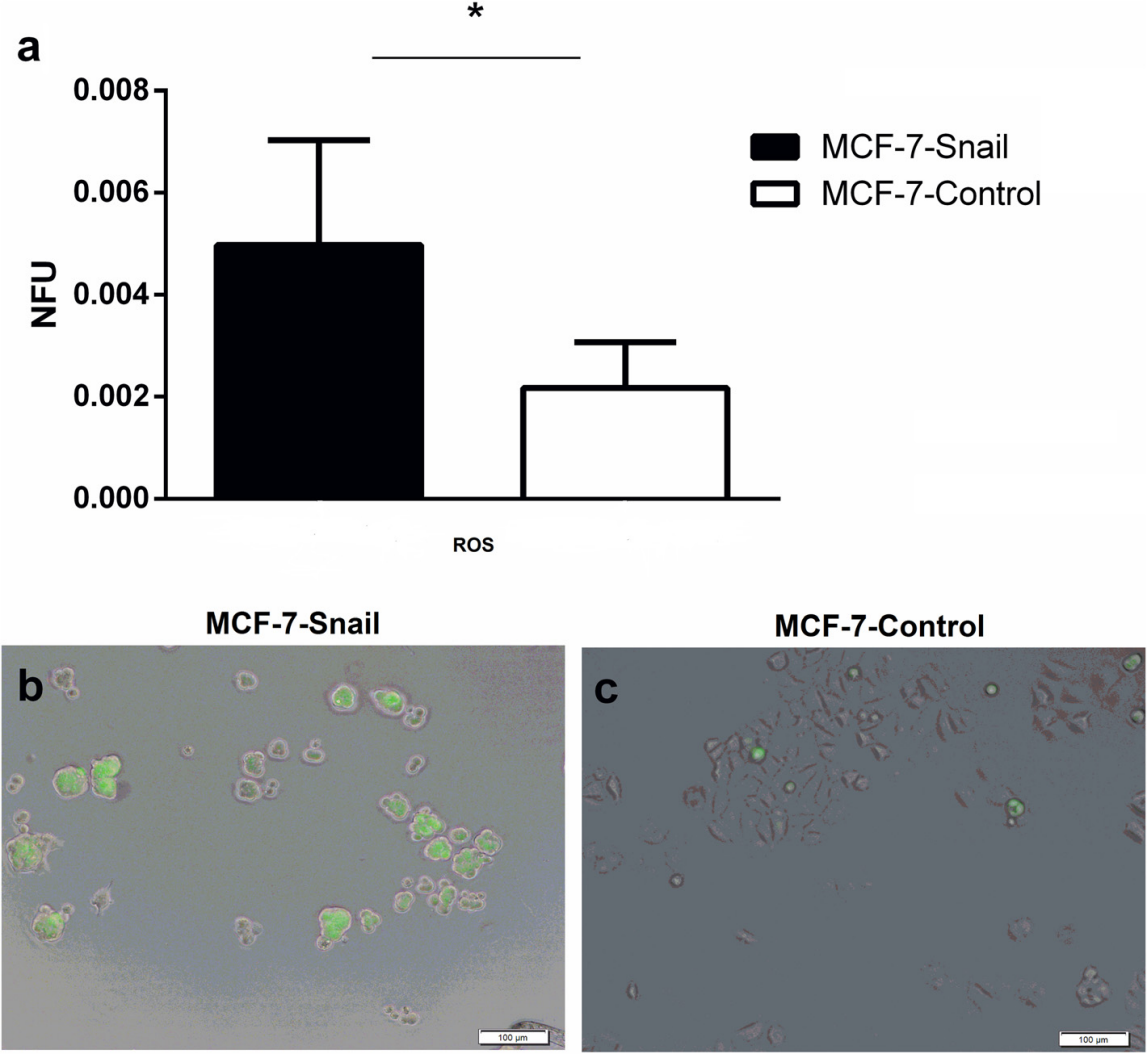
The level of ROS detected by H2DCF-DA staining in *MCF-7-Snail* vs *MCF-7-Control* cells. Levels of ROS detected by fluorimetry (**a**) and representative epifluorescence microscopy images overlaid on brightfield images (**b**: *MCF-7-Snail*, **c**: *MCF-7-Control* cells). *NFU* normalized fluorescence units, Error bars: SD; *p*-value = 0.0471 (two-tailed t-test); scale bar: 100 μm (*: *p* <0.05)

Reactive oxygen species (ROS) are known to induce a variety of cellular responses contributing to the development and progression of breast and other types of cancer [43]. Since our systems analyses indicated that ROS-associated cellular process networks are enriched by genes differentially expressed between *MCF-7-Snail* and *MCF-7-Control* cells (Table 2), we explored the possibility that the ectopic expression of *Snail* in *MCF-7* cells might be associated with changes in levels of ROS contributing to the process of EMT and other *Snail*-mediated phenotypic changes in *MCF-7-Snail* cells. The results, presented in Fig. 7, are consistent with this hypothesis and demonstrate significantly higher levels of ROS in mesenchymal-like *MCF-7-Snail* cells relative to the *MCF-7-Controls*. These results are also consistent with recently reported findings in mesenchymal-like prostate cancer cells [44].

### Elevated levels of ROS in *MCF-7-Snail* cells are associated with decreased levels of cellular glutathione and NF-κB activity

**Fig. 8:**
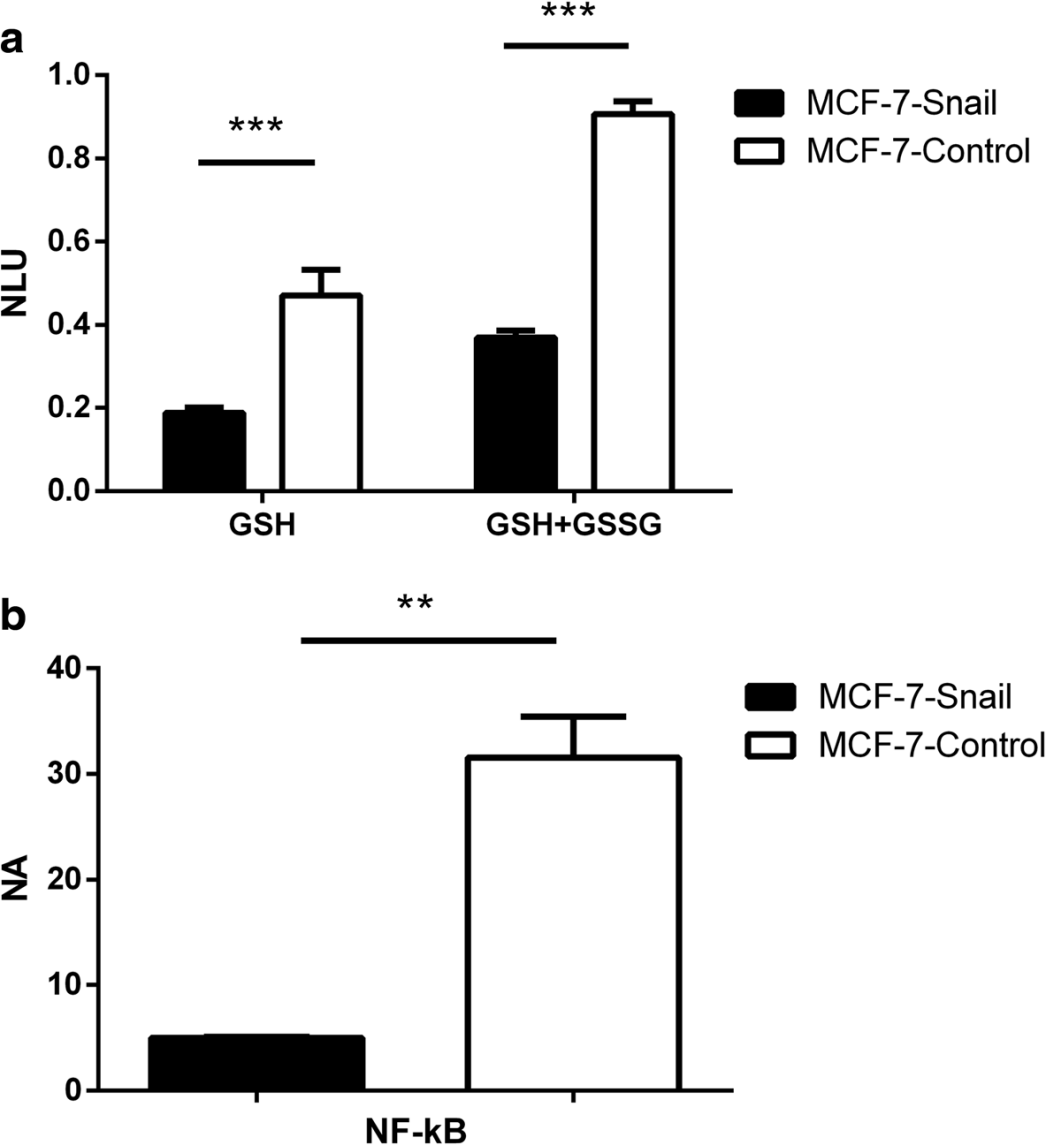
Levels of GSH, GSH+GSSG and nuclear NF-κB. **a** Levels of free (GSH) and total (GSH+GSSG) glutathione in *MCF-7-Snail* and *MCF-7-Control* cells determined by luminescent assay; *NLU* normalized luminescence units; *p*-values (multiple t-test with Holm-Šidák correction): GSH = 9.98 × 10^−5^; GSH+GSSG = 8.46 × 10^−8^. **b** Levels of nuclear NF-κB in *MCF-7-Snail* and *MCF-7-Control* cells expressed as determined by ELISA in nuclear protein lysates; *NA* normalized absorbance; Error bars: SD; *p* = 0.0071 (Welch’s corrected t-test; **: *p* <0.01; ***: *p* <0.001)

Increased intracellular concentrations of ROS in cancer cells have been previously associated with decreased levels of antioxidant enzymes and/or glutathione [45]. We compared intracellular levels of glutathione in *MCF-7-Snail* and *MCF-7-Control* cells using a luminescence-based assay. The results presented in Fig. 8a demonstrate lower levels of both reduced (GSH) and total (GSH+GSSG) glutathione levels in *MCF-7-Snail* cells relative to *MCF-7-Controls*.

ROS have been shown to induce EMT in mouse mammary epithelial cells via NF-κB-mediated activation of *Snail* [46]. In addition, NF-κB was reported to play an essential role in the induction and maintenance of EMT in breast cancer [47]. Given the previously observed associations between the ROS and NF-κB activity in breast and other cancer types [48], we compared the status of NF-κB activity in *MCF-7-Snail* and *MCF-7-Control* cells. Our microarray data indicate down regulation of the *NF-κB1* gene in *MCF-7-Snail* cells (Additional files 3 and 4). In addition, we found that the NF-κB transcription network is enriched by genes differentially expressed between *MCF-7-Snail and MCF-7-Control* cells (Additional file 6). NF-κB transcript levels do not necessarily correlate with NF-κB activity since the protein is activated by proteasome degradation of IκB, and the subsequent translocation of NF-κB dimers from the cytoplasm to the nucleus [49]. Thus, measurement of NF-κB protein levels in cell nuclei is necessary for biologically accurate estimates of NF-κB activity. We determined the level of NF-κB (p50/p65- the most common member of NF-κB/Rel family) by its quantification in nuclear protein extracts using an ELISA-based method. The results, presented in Fig. 8b, demonstrate a significant decrease in nuclear protein levels of NF-κB in *MCF-7-Snail* cells relative to controls indicating decreased nuclear translocation and activation.

### The consequence of ectopic expression of *Snail* on drug sensitivity is variable

**Fig. 9:**
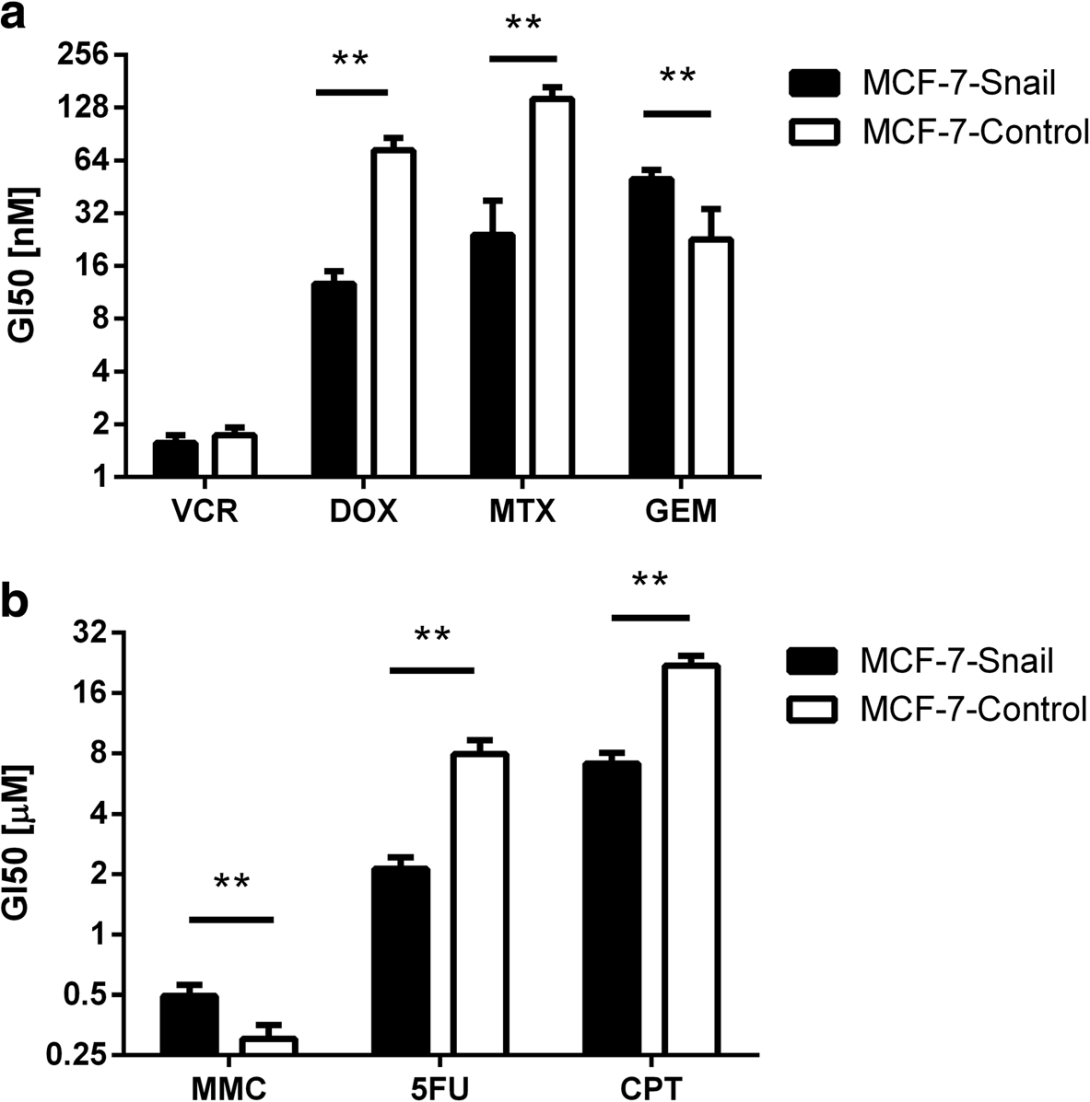
Drug sensitivity for *MCF-7-Snail* and *MCF-7-Control* cells determined by Tox-8 assay and expressed as GI50 values. **a** vincristine (VCR), doxorubicin (DOX), methotrexate (MTX), gemcitabine (GEM); **b** mitomycin C (MMC), 5-fluorouracil (5FU), cisplatin (CPT). Error bars: SD; *N* = 4 replicates. Statistical significance of differences between mean GI50 values determined by t-test corrected by Holm-Šidák method (**: *p* <0.01)

Since EMT has been reported by several groups to enhance the resistance of cancer cells to anticancer drugs (reviewed in [9]), we evaluated the relative sensitivity of *MCF-7-Snail* and *MCF-7-Control* cells to a variety of cancer drugs previously and/or currently employed in breast cancer treatment (vincristine, doxorubicin [50], mitomycin C, methotrexate [51], gemcitabine, cisplatin [52] and 5-fluorouracil [53]). The sensitivity assay was designed to compare numbers of viable cells in drug-treated cell cultures relative to untreated controls independent of the specific mechanisms of cell cytotoxicity. GI_50_ values for each drug and cell type (Fig. 9) were determined from dose response curves (Additional file 2: Figure S7).

The results indicate that mesenchymal-like *MCF-7-Snail cells* are significantly more sensitive to doxorubicin, methotrexate, cisplatin, and 5-fluorouracil relative to epithelial-like *MCF-7-Control* cells. Conversely, *MCF-7-Snail* cells display significantly reduced sensitivity to gemcitabine and mitomycin C. While the difference in GI_50_ values between the two cell types is statistically significant for MMC, the fold change (~1.6 ×) is relatively low and, thus, of questionable biological significance. *MCF-7-Snail* and *MCF-7-Control* cells did not display significant differences in sensitivity to vincristine.

**Fig. 10:**
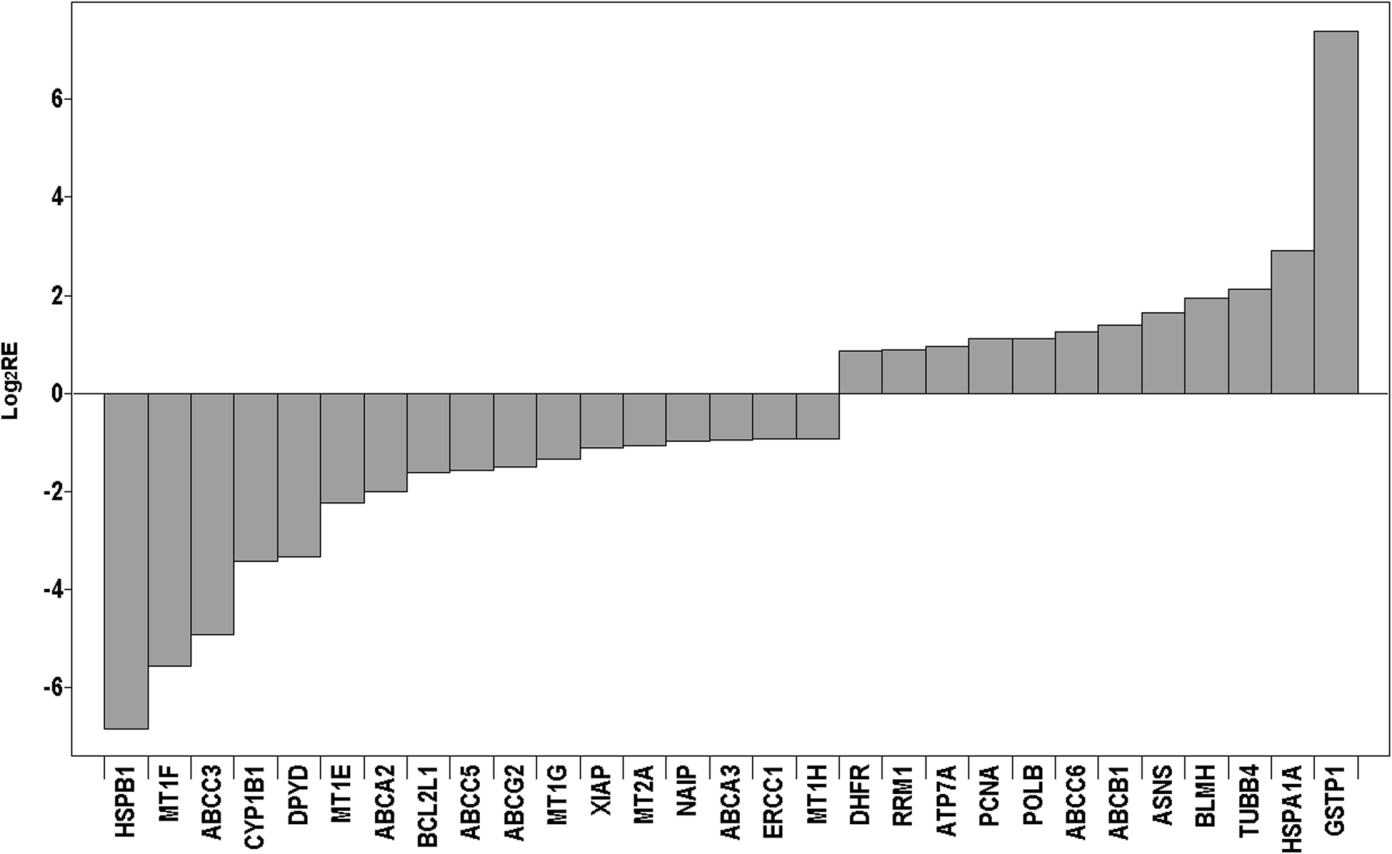
Relative expression of a subset of 53 drug resistance-related genes that displayed significantly different expression in *MCF-7-Snail* relative to *MCF-7-Control* cells. Relative expression from microarray data is presented in log2 scale

Differences in sensitivity of *MCF-7-Snail* and *MCF-7-Control* cells to some anticancer drugs tested in this work can be interpreted in the context of changes in the expression of genes known to contribute to resistance against specific anticancer drugs (Fig. 10). For example, increased sensitivity of *MCF-7-Snail* cells to cisplatin can be attributed to the combined effect of (i) down regulation of cisplatin-sequestering metallothioneins MT1E, MT1F and MT1G [54], (ii) down regulation of the ABCC3 transporter [55], down regulation of the nucleotide excision repair enzyme ERCC1 [56], as well as (iii) down regulation of the anti-apoptotic protein XIAP [57], and (iv) decreased concentration of GSH (Fig. 8a). In fact, the lower concentration of GSH in *MCF-7-Snail* cells likely suppressed the effect of up regulation of GSTP1 in *MCF-7-Snail* cells that would otherwise contribute to the resistance against cisplatin [58]. Likewise, increased sensitivity of *MCF-7-Snail* cells to doxorubicin could be attributed to the down regulation of HSPB1 [59] and metallothioneins [60], while increased sensitivity to 5-FU can be attributed to the down regulation of DPYD in *MCF-7-Snail* cells [61]. On the other hand, lower sensitivity of *MCF-7-Snail* cells to gemcitabine can be attributed to the up regulation of its target, RRM1 [62]. Overall, these results indicate that EMT is not always associated with increased resistance to anticancer drugs.

### *MCF 7-Snail* cells display increased radiosensitivity relative to *MCF-7-Controls*

**Fig. 11:**
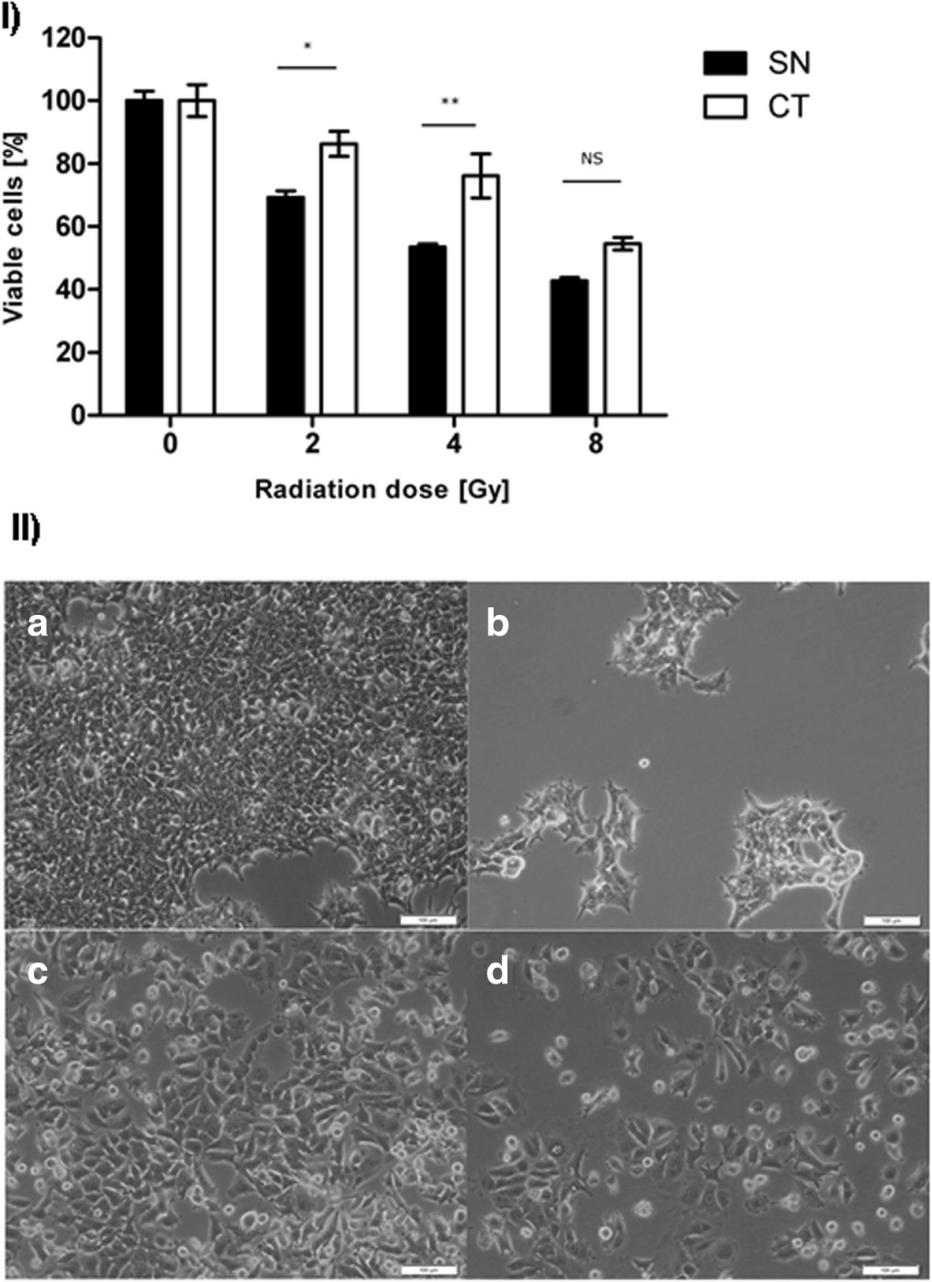
Radiation sensitivity for *MCF-7-Snail* and *MCF-7-Control* cells. **I** Radiation sensitivity determined from the number of viable cells 72 h post-irradiation by specified doses of X-ray. Viable cells [%] corresponds to the number of viable cells determined by Tox-8 assay in treatment relative to non-irradiated control cultures (*N* = 2 replicates; *: *p* <0.05; **: *p* <0.01); error bars = SD. **II** Micrographs of *MCF-7-Snail* (*a, b*) and *MCF-7-Control* cells (*c, d*) 72 h post-irradiation with 0 Gy (*a, c*) or 4 Gy (*b, d*). Scale bar: 100 μm. SN = *MCF-7-Snail* cells; CT = *MCF-7-Control* cells

Radiotherapy is an important treatment modality for breast cancer [63]. The efficacy of radiotherapy, however, can be decreased by intrinsic or acquired resistance [64] possibly associated with EMT [8, 65]. To explore this relationship in our experimental system, we exposed *MCF-7-Snail* and *MCF-7-Control* cells to a single dose of X-ray radiation at 2–8 Gy and cells were allowed to replicate for 72 h at which point the number of metabolically active viable cells was determined. The results demonstrate a significantly lower proportion of viable *MCF-7-Snail* cells at 72-hours post-irradiation (Fig. 11) indicating higher radiation sensitivity of the mesenchymal-like *MCF-7-Snail* cells relative to epithelial-like *MCF-7-Control* cells. Consistent with the response to radiation treatment, we also observed prominent multinucleated cells in cultures of irradiated *MCF-7-Snail* cells (Fig. 11) indicative of radiation-induced deregulation of mitosis and deficient separation of nuclei during cytokinesis [66, 67].

**Fig. 12:**
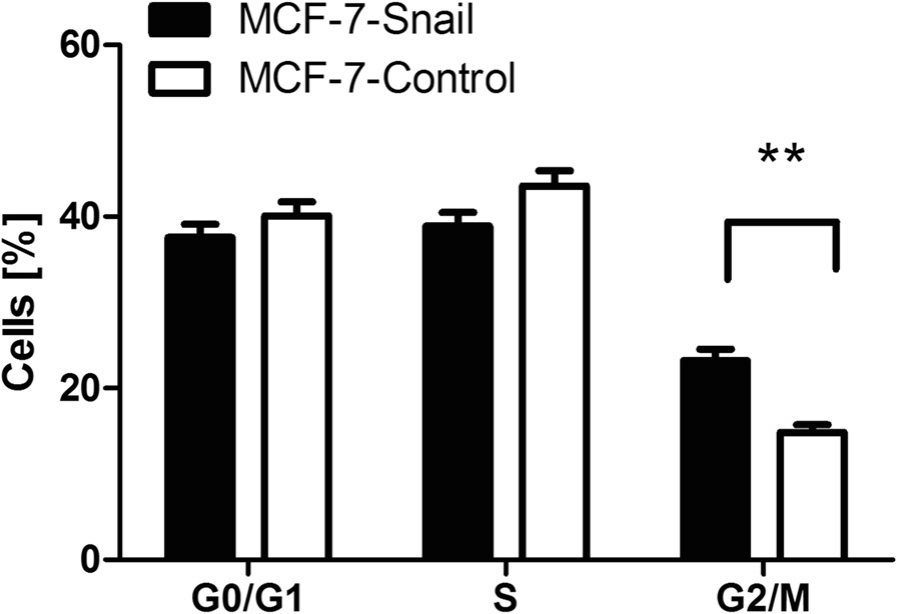
Cell cycle distribution of *MCF-7-Snail* and *MCF-7-Control* cells at the time of X-ray irradiation. (means ± SEM; *N* = 3 replicates; **: *p* <0.01)

Since radiosensitivity has previously been shown to be dependent on the phase of the cell cycle [68], we examined the distribution of *MCF7-Snail* and *MCF7-Control* cells in specific phases of the cell cycle before irradiation. Cultures of *MCF-7-Snail* and *MCF-7-Control* cells plated at densities identical to those used in the radiation sensitivity experiments displayed a significantly higher proportion of *MCF-7-Snail* cells in the G2/M phase relative to *MCF-7-Control* cells (Holm-Šidák corrected t-test *p* = 0.0059; CI95 = 2.483–14.29 %). Differences in the proportion of the two cell types in G0/G1 and S phases of the cell cycle were not significant (Fig. 12 and Additional file 2: Figure S8). Since cells are known to display their greatest sensitivity to irradiation during mitosis and G2 phases of the cell cycle [69], the higher proportion of *MCF-7-Snail* cells in the G2/M phase of the cell cycle may contribute to their higher radiosensitivity relative to *MCF-7-Control* cells.

However, different slopes of semi-log radiosensitivity plots at a medium-high dose range (Additional file 2: Figure S9) suggest that the observed difference in the radiosensitivity between *MCF-7-Snail* and *MCF-7-Control* cells cannot be attributed only to the cell cycle differences. In an initial effort to identify molecular differences potentially responsible for the observed differences in radiation sensitivity of these two cell types, we focused on genes previously associated with radiation resistance in breast and other cancers and found that many of them were significantly down regulated in *MCF-7-Snail* cells (Additional file 4), e.g. *IGF1R* (insulin-like growth factor one receptor) [70], *REG4* (Regenerating islet-derived protein four) [71], *RALBP1* (RalA-binding protein one) [72] and *ERCC1* (excision repair cross-complementation group one) [73]) suggesting their potential involvement in the increased sensitivity of *MCF-7-Snail* cells to radiation treatment.

## Discussion

An abundance of prior evience indicates that breast cancer progression and the development of MBC is intimately associated with EMT [74–76]. In addition, EMT has also been associated with the resistance of breast and other cancers to a variety of anticancer drugs [9] and ionizing radiation [8, 65]. While dysregulation of the transcription factor *Snail* has been previously associated with EMT in breast and other cancers [10, 12], its effect on system-wide molecular changes and consequent changes in response to radiation and drug therapies is incompletely understood. In an effort to better understand the role of *Snail* in breast cancer development, we examined an isogenic pair of breast cancer cell lines *MCF-7-Snail* and *MCF-7-Control. MCF-7-Snail* cell line has been previously engineered to ectopically express *Snail* [14]. Detailed anayses of *MCF-7-Snail* cells provides a unique opportunity to evaluate biologically and clinically significant consequences of *Snail*-induced EMT in breast cancer.

Previous studies have demonstrated that *MCF-7-Snail* cells display reduced expression of the epithelial marker E-cadherin (CDH1), increased expression of the mesenchymal markers VIM and FN1 and enhanced migratory capabilities relative to controls [14]. Consistent with these earlier findings, our microarray analyses of *MCF-7-Snail* cells demonstrate concerted changes in the expression of a number of genes and pathways previously implicated in EMT [25]. Among the genes significantly up regulated/activated in response to ectopic *Snail* expression are several key transcription factors known to be critical to EMT development (reviewed in [77]). For example, the initial stages of EMT are believed to require *Snail*-induced repression of E-cadherin, while subsequent maintenance may require cooperation of other key transcription factors, including Slug, E47, Zeb2 and Twist [77]. Consistent with these expectations, we observed significant down regulation of E-cadherin and up regulation of Slug, E47, Zeb2, and Twist 1 in *MCF-7-Snail* relative to *MCF-7-Control* cells.

Snail and Slug are believed to work cooperatively in EMT [77–79]. Our results suggest that the mechanism for *Snail*-mediated up regulation of *Slug* is through the down regulation of estrogen receptor 1 (ESR1). *Snail* is a known transcriptional repressor of *ESR1* [80] that, in turn, is a documented transcriptional repressor of *Slug* [81]. Consistent with this interactive model, we observed a significant up regulation of *Slug* and concurrent down regulation of *ESR1* in *MCF-7-Snail* cells relative to controls. Interestingly, this down regulation of *ESR1* was found be correlated with other molecular changes indicative of *Snail*-induced transformation of *MCF-7* cells from the luminal A to the clinically more aggressive triple-negative, claudin-low sub-type. This finding is consistent with the potential of *Snail*-targeted therapy for the treatment of metastatic breast cancer [82].

Interactions between *Snail* and *Slug* may also contribute to our observed down regulation in the expression of the miR-200 family of microRNAs in *MCF-7-Snail* cells. In agreement with prior studies implicating members of the miR-200 family of microRNAs with EMT/MET (mesenchymal-to-epithelial transition) [28, 32, 33], we observed a significant down regulation of members of the miR-200 family in *MCF-7-Snail* cells. Our gene expression analyses suggest several possible mechanisms by which ectopic expression of *Snail* in *MCF-7* cells may be contributing to repression of miR-200 family of miRNAs.

Although *Snail* has been previously implicated as an activator of Zeb1 [83] and Zeb2 [84], both of which are well-established transcriptional repressors of miR-200 family members [85], Snail-activation of Zeb1/2 is believed not to be direct [83, 86]. In contrast, *Slug* is known to be a direct transcriptional activator of Zeb1 [86] and can itself directly repress transcription of miR-200 family members [87]. Thus, *Snail*-induced activation of *Slug* in *MCF-7-Snail* cells may contribute to the down regulation of miR-200 family microRNAs.

Our results also demonstrate a significant down regulation of the transcription factor Krüppel-like factor 5 (KLF5) in *MCF-7-Snail* cells (Additional files 3 and 4). KLF5 is a known transcriptional activator of miR-200 family members, as well as several other microRNAs previously implicated in EMT (e.g.*,* miR-205 and Let-7a/7b/7c/7d/7e/7g) [88]. It has been previously suggested that KLF5-Smads-p300 complexes activate while ZEB1/2-Smads-p300 complexes repress the transcription of miR-200, and that KLF5 and ZEB1/2 may physically interact or compete to execute opposing functions in miR-200 regulation [88].

A third possible mechanism of miR-200 family down regulation suggested by our results involves the nuclear receptor PELP1 and the histone deacetylase HDAC2. Both of these genes were found to be significantly up regulated in *MCF-7-Snail* cells (Additional files 3 and 4). It has recently been shown that PELP1, in cooperation with histone deacetylase HDAC2, can transcriptionally repress mir-200 family microRNAs [89]. Interestingly, we also found that MYC, a known transcriptional activator of PELP1 [90], is functionally activated in *MCF-7-Snail* cells. None of the above possible modes of miR-200 family repression are mutually exclusive and suggest a redundant and highly interactive nature of *Snail* regulatory controls.

Previous studies have implicated *Snail*-induced activation of TGF-β with EMT in breast cancer cells [91, 92]. While our pathway enrichment analysis identified “TGF-beta-dependent induction of EMT via SMADs” as a significantly enriched pathway (Table 1), this does not necessarily imply TGF-β activation. In fact, our gene expression analysis indicates down-regulation of TGFB1-3, SMAD2 and SMAD3 genes in *MCF-7-Snail* cells (Additional files 3 and 4) which argues against activation of TGF-β mediated signaling via receptor-regulated Smads [93]. In addition, we failed to observe up regulation of known downstreatm targets of TGF-β (e.g., CTGF, ligand TGFB2 and receptor TGFBR2) in *MCF-7-Snail* cells, again suggesting the absence of TGF-β-signaling. Consistent with these findings, previous studies of claudin-low breast cancer subtypes indicate that Snail-induced EMT need not be dependent on TGF-β-mediated autocrine signaling [92].

Several previous studies have noted an interactive relationship between changes in the expression of key regulatory genes and intracellular levels of ROS in EMT [44, 46, 94]. This regulatory relationship coupled with their association with oxidative damage and chronic inflammation has identified ROS as a major contributing factor in the progression of breast and other cancers [43, 94]. Our finding that ROS levels are significantly elevated in *MCF-7-Snail* cells is consistent with these and other results indicating that elevated levels of ROS contribute to EMT and the maintenance of the mesenchymal phenotype in metastatic cancer cells.

Several studies have previously correlated increasing levels of ROS with activation of the transcription factor NF-κB, possibly as part of the stress response pathway [48]. For example, ROS were shown to activate NF-κB that subsequently induced the expression of Snail in MMP3-mediated EMT of mammary epithelial cells [46]. In contrast to expectations, we observed a significant decrease in the expression of NF-κB1 in *MCF-7-Snail* cells relative to controls. We also observed a decrease in nuclear protein levels of *NF-κB* in *MCF-7-Snail* cells indicating reduced NF-κB activation in these cells. Collectively, our results indicate that *Snail*-induced increases in intracellular levels of ROS are not coupled with NF-κB activation in *MCF-7-Snail* cells. The reduced activation of NF-κB in *MCF-7-Snail* cells may be associated with glutathione depletion, consistent with a previously report that demonstrated IκB kinase-dependent and independent down-regulation of NF-κB activity upon glutathione depletion [95].

In addition to a systems analysis of the molecular consequences of *Snail*-induced EMT in *MCF-7* cells, we were also interested in documenting the effect of these molecular changes on drug and radiation sensitivity. A number of prior studies have suggested that EMT is associated with increased resistance of cancer cells to anticancer drugs (reviewed in [9]). However, our findings suggest that this generalization may not be universally correct. While we found that *MCF-7-Snail* cells are significantly more resistant to gemcitabine and mitomycin C (MMC), they were significantly more sensitive to doxorubicin, methotrexate, cisplatin, and 5-fluorouracil. Similar inconsistencies between EMT and drug sensitivities have been previously reported [96–98]. For example, in a survey of the sensitivity of 54 adherent human cancer cell lines from the NCI-60 cell panel, statistically significant positive correlations between expression levels of the epithelial biomarker E-cadherin and drug sensitivity was found for only ten out of 118 drugs [96]. In another study, non-small cell lung cancer (NSCLC) cells designated as mesenchymal-like based on their molecular profile, were found to be more resistant than epithelial-like NSCLC cells to erlotinib and the PI3K/AKT/mTOR inhibitors GDC0941 and 8-aminoadenosine, but more sensitive to pemetrexed, paclitaxel and docetaxel [97]. Similarly, two mesenchymal-like pancreatic cancer cell lines were found to display greater sensitivity to paclitaxel than epithelial-like pancreatic cancer cell lines [98]. Collectively, these findings indicate that epithelial-mesenchymal cellular phenotypes, per se, are not reliable predictors of relative drug sensitivity. While such inconsistencies may simply be attributable to other differences between cell lines that are not consistently associated with epithelial or mesenchymal phenotype, it may also be a reflection of a continuum of potential EMT-mediated effects on cellular phenotypes [99].

Another reflection of the inconsistency of EMT-mediated effects on cellular phenotypes is our finding that mesenchymal-like *MCF-7-Snail* cells are more sensitive to radiation treatment than their parental epithelial-like cells. In contrast to our results, a number of previous studies have associated EMT and the mesenchymal cell phenotype with increased radioresistance [8, 65]. For example, epithelial ovarian cancer cells, in which the ectopic expression of Snail or Slug resulted in the acquisition of stem cell-like properties, were found to be associated with radioresistance [65]. Likewise, mesenchymal-like breast cancer cells that displayed higher resistance to radiation than their parental epithelial-like cells, were also found to exhibit a stem cell-like phenotype including a CD44^+^/CD24^-/low^ molecular signature [100]. One possible explanation of the apparent discrepancy between our results and the accumulated evidence that mesenchymal-like cancer cells are generally associated with radiation resistance (reviewed in [101]) is that it is the stem cell-like properties of these cells and not EMT per se that is responsible for their radiation resistance. While stem cells typically display a mesenchymal-like phenotype, not all EMT-induced mesenchymal-like cells may display features characteristic of stem cells. For example, it has been previously shown that cancer stem cells typically contain lower levels of ROS than their more differentiated progeny and that this difference is critical for the maintenance of stem cell phenotype [102]. In contrast, we found that *MCF-7-Snail* cells contain higher levels of ROS than *MCF-7-Control* cells. In addition, *MCF-7-Snail* cells display down regulation of Sox2 and Nanog (Additional files 3 and 4) that have been previously shown to contribute to the maintenance of pluripotency and the self-renewal properties of human embryonic stem cells [103]. In addition, we found that other stem cell biomarkers are also down regulated or unchanged in expression in *MCF-7-Snail* cells relative to controls (e.g., UTF1 [104], FBXO15 [105], ALDH1A3 [106], CD44, CD133 and ITGB1 [107]). Furthermore, previous studies have also reported down-regulation of several genes associated with stem cell phenotype early (ABCG2 [108], CD44, ALDH1A3) or late (SOX9 [109]) after induction of Snail expression in immortalized human mammary epithelial MCF10A cells [15]. Collectively, these findings indicate that *MCF-7-Snail* cells are not stem cell-like cells and that EMT per se does not necessarily lead to the acquisition of resistance to radiation and anticancer drugs.

## Conclusions

Constitutive ectopic expression of *Snail* in the epithelial-like, luminal A-type, breast cancer cell line *MCF-7* results in significant changes in the expression >7600 genes including master gene and miRNA regulators of EMT. Ectopic expression of Snail induced *MCF-7* cells to undergo EMT and to acquire features characteristic of triple-negative, claudin-low breast cancer cells but not of breast cancer stem-like cells. *Snail*-induced EMT of *MCF-7* cells resulted in increased sensitivity to radiation treatment but increased, decreased or no change in sensitivity to a variety of anticancer drugs indicating that EMT is not necessarily predictive of decreased responsiveness to therapeutic treatments. These results underscore the complexity and cell-context dependent nature of EMT-mediated changes in breast cancer cells.

### Availability of supporting data

A detailed description of the microarray experiment and the resulting data are available in the Gene Expression Omnibus repository (GEO, http://www.ncbi.nlm.nih.gov/geo/) under the accession number GSE58252.

## Additional files

Additional file 1: List of 71 genes relevant to EMT and 53 genes relevant to anticancer drug resistance assembled by manual curation from appended references. (XLSX 54 kb) Additional file 2: Supplemental Figures S1-S9, supplemental tables S1 and S2 and supplemental methods S1-S3. (PDF 528 kb) Additional file 3: List of 12,104 probe sets corresponding to differentially expressed transcripts between *MCF-7-Snail* and *MCF-7-Control* cells identified by Significance Analysis of Microarray (FDR = 2.12 % and |FC| ≥1.5). (XLSX 2199 kb) Additional file 4: List of 7602 unique annotated genes differentially expressed between *MCF-7-Snail* and *MCF-7-Control* cells. (XLSX 178 kb) Additional file 5: List of 164 network objects from the MetaCore human protein interactome that are statistically significantly over-connected to the genes differentially expressed between *MCF-7-Snail* and *MCF-7-Control* cells. (XLS 71 kb) Additional file 6: List of 31 significant networks centered on transcription factors built from genes differentially expressed between *MCF-7-Snail* and *MCF-7-Control* cells. (XLS 39 kb) Additional file 7: List of differentially expressed genes between *MCF-7-Snail* and *MCF-7-Control* cells known to MetaCore knowledgebase to be directly transcriptionally regulated by c-Myc. (XLS 610 kb) Additional file 8: List of differentially expressed genes between *MCF-7-Snail* and *MCF-7-Control* cells known to MetaCore knowledgebase to be directly transcriptionally regulated by ESR-1. (XLS 339 kb) Additional file 9: List of gene sets from C2: Curated Gene Sets and C6: Oncogenic Signatures gene sets (http://www.broadinstitute.org/gsea/msigdb/collections.jsp) significantly enriched in *MCF-7-Snail* or *MCF-7-Control* phenotypes. (XLSX 24 kb)

## Abbreviations

CSC: cancer stem-like cells
DOX: doxorubicine
EMT: epithelial-to-mesenchymal transition
5-FU: 5-fluorouracil
GEM: gemcitabine
GSH: glutathione (reduced form)
GSSG: glutathione (oxidized disulfide form)
H2DCF-DA: 2′,7′-dichlorodihydrofluorescein diacetate
MBC: metastatic breast cancer
MMC: mitomycin C
MTX: methotrexate
NSCLC: non-small cell lung cancer
ROS: reactive oxygen species
VCR: vincristine
