# Supplementary material for: Snail-induced epithelial-to-mesenchymal transition of MCF-7 breast cancer cells: systems analysis of molecular changes and their effect on radiation and drug sensitivity
Source: BMC Cancer. 2016 Mar 18;16:236. doi: 10.1186/s12885-016-2274-5 (PMC4797178; doi:10.1186/s12885-016-2274-5)
Supplement: Additional file 2: — Supplemental Figures S1-S9, supplemental tables S1 and S2 and supplemental methods S1-S3. (PDF 528 kb) [file 12885_2016_2274_MOESM2_ESM.pdf]

Mezencev R. et al. ***Snail*-induced epithelial-to-mesenchymal transition of *MCF-7* breast cancer cells: systems analysis of molecular changes and their effect on radiation and drug sensitivity**

Additional file 2:

Supplemental figures

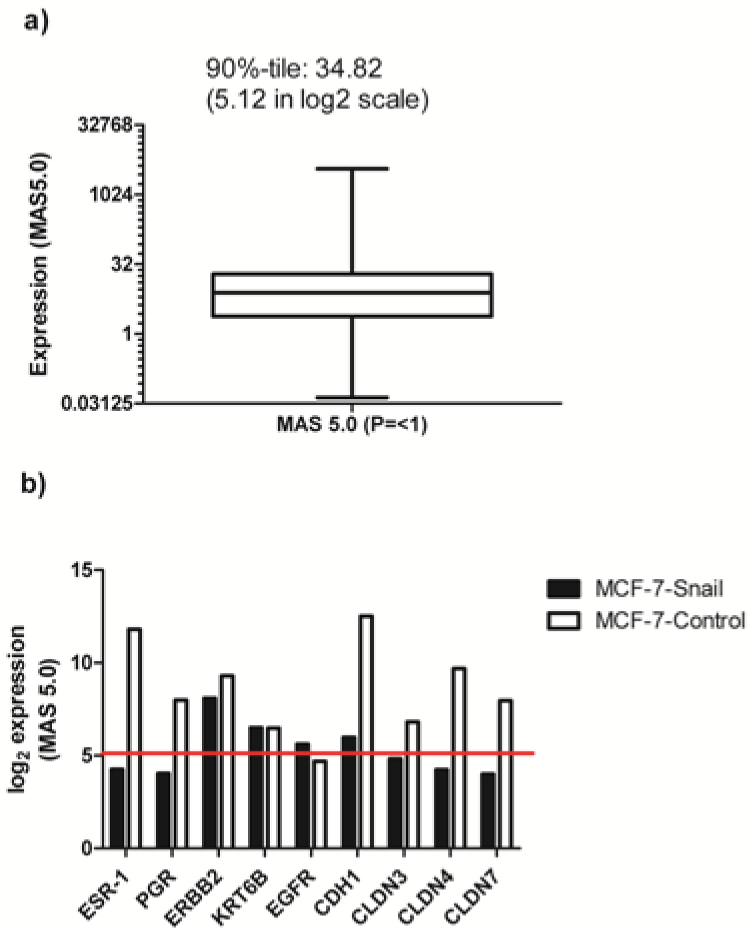

Figure S1 (a) Distribution of MAS 5.0 signals for probe sets with 5 or more absent calls across 6 specimens to determine 90%-tile as a threshold to identify genes that are likely "not-expressed"; (b) Applying threshold to MAS 5.0 normalized data confirms "not-expressed" status for ESR-1, PGR, CLDN3, CLDN4 and CLDN7 genes in MCF-7-Snail cells. Probe sets for ERBB2, CDH1 and CLDN3 (type:_s_at) and KRT6B (type: x_at) are not specific and may detect other transcripts. Known negativity of MCF-7 cells for ERBB2 and KRT6B [1] supports the negative status of MCF-7-Snail cells for these markers as well.

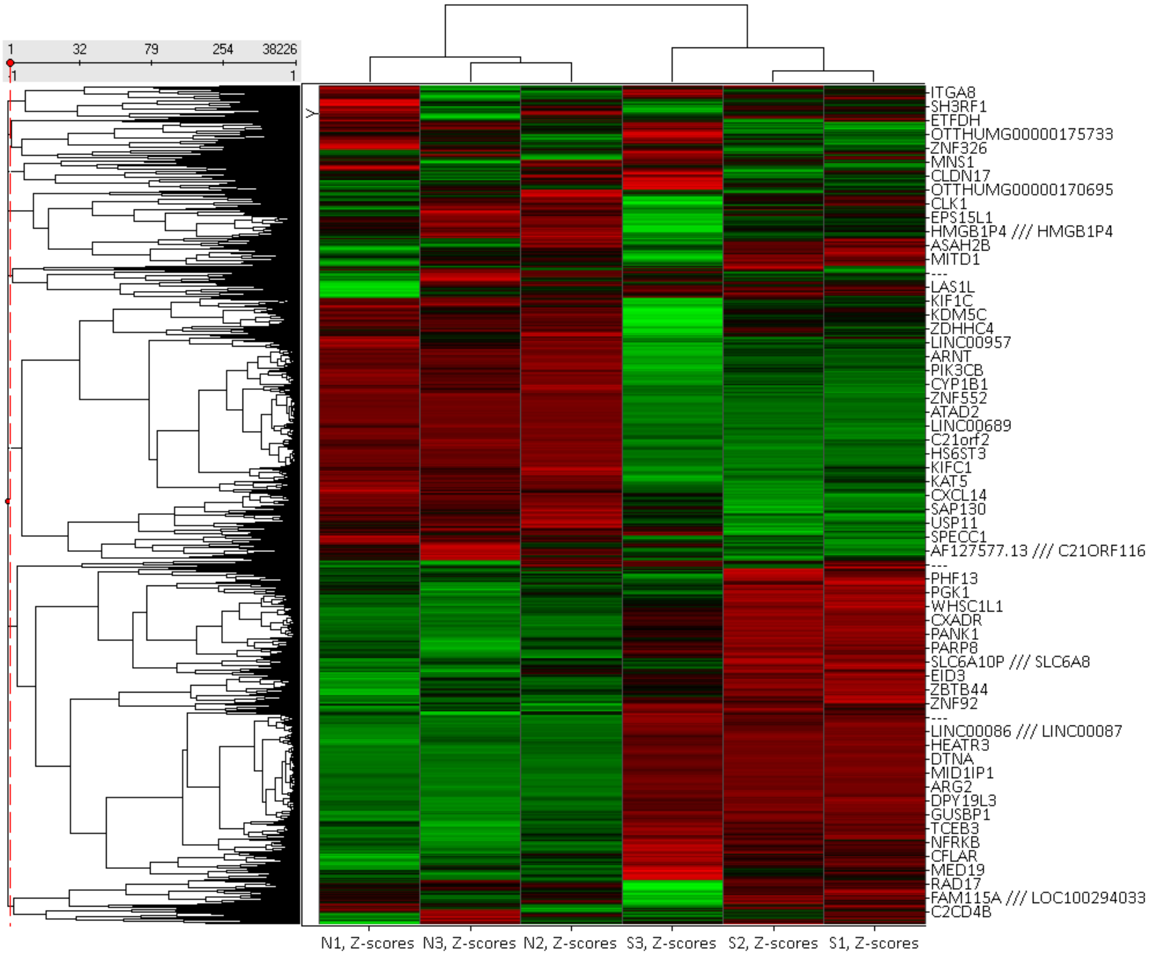

Figure S2 Hierarchical clustering of Z-score normalized expression data (PLIER+16) for 38,226 probe sets (at least one call was not “A” across all 6 specimens). Clustering method: Complete linkage (maximum); Similarity measure: Correlation; Ordering function: Average value. N1-N3: MCF-7-Control cells; S1-S3: MCF-7-Snail cells. Color coding: green – z-score < 0; red – z-score > 0

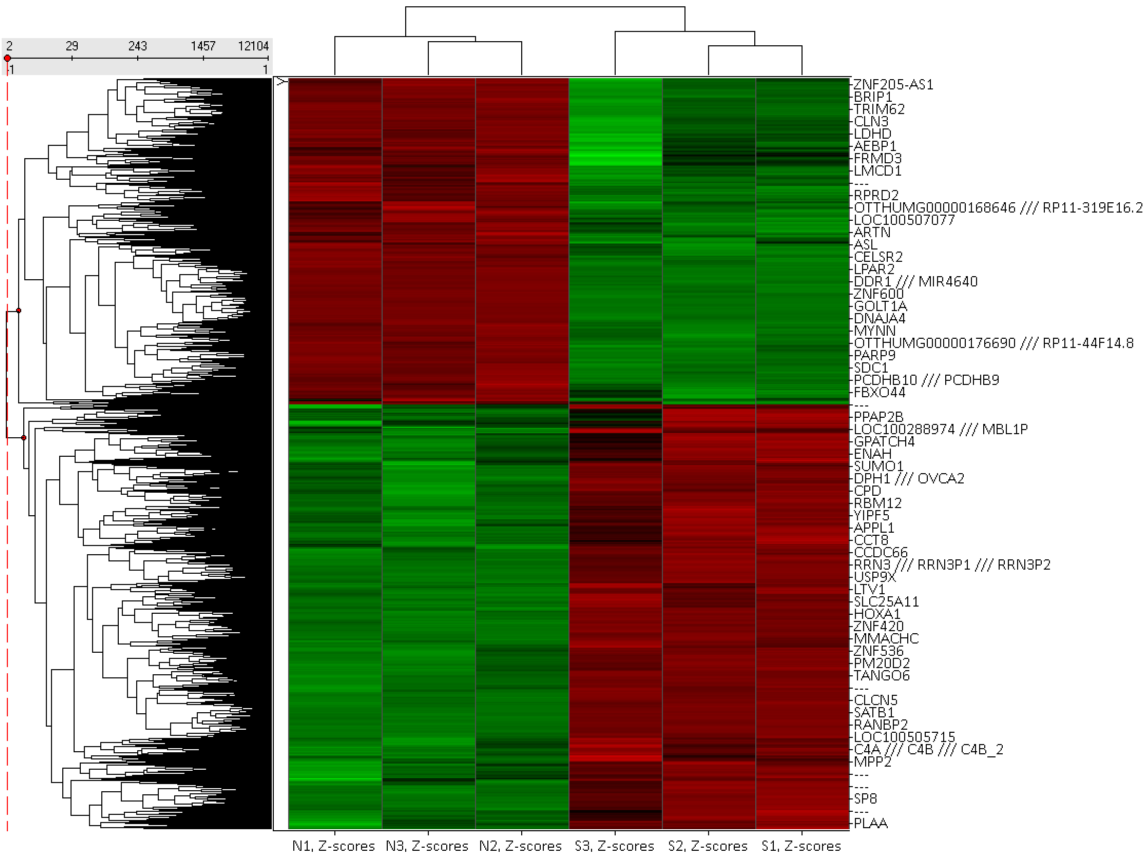

Figure S3 Hierarchical clustering of Z-score normalized expression data (PLIER+16) for 12,104 probe sets that detected differentially expressed transcripts between MCF-7-Snail and MCF-7-Control cells. Clustering method: Complete linkage (maximum); Similarity measure: Correlation; Ordering function: Average value. N1-N3: MCF-7-Control cells; S1-S3: MCF-7-Snail cells.

Color coding: green – z-score < 0; red – z-score > 0

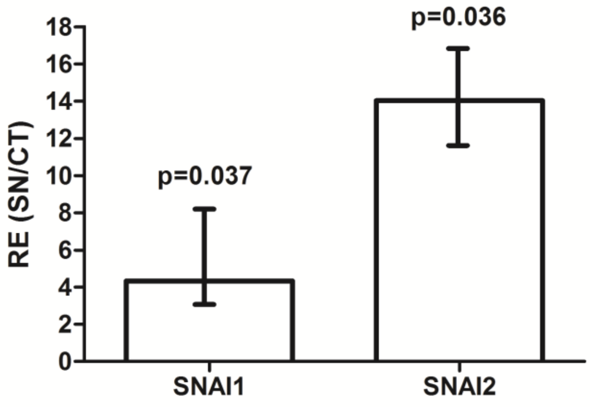

Figure S4 Relative expression of Snail (SNAI1) and Slug (SNAI2) in *MCF-7-Snail* vs. *MCF-7-Control* cells. Relative expression (RE) was determined by qPCR (SYBR Green, see Supplemental method S1). Error bars: 95% CI (N=3 replicates).
p-values were calculated by randomization test.

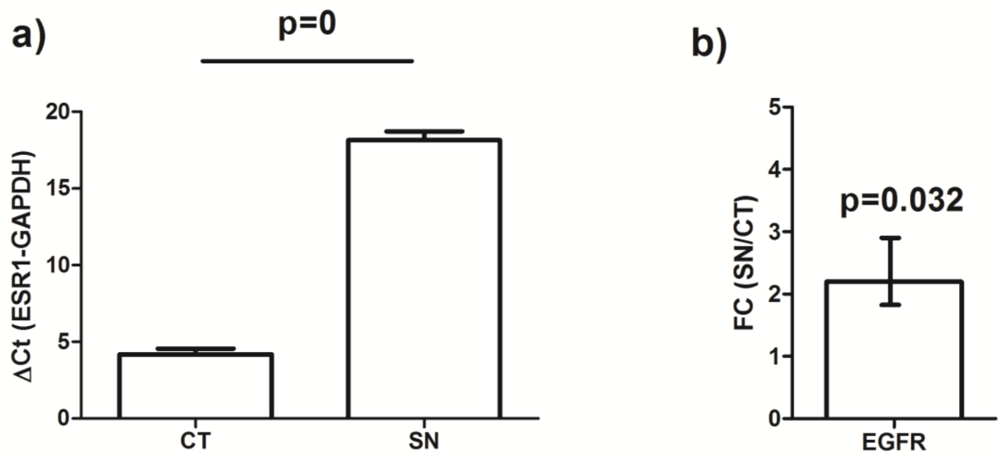

Figure S5 Relative expression of ESR1 (a) and EGFR (b) in *MCF-7-Snail* (SN) vs. *MCF-7-Control (CT)* cells (b) determined by qPCR (TaqMan, see Supplemental method S2). (a) Results are presented as mean normalized threshold cycle values due to very low relative expression of ESR1 in MCF-7-Snail vs MCF-7-Control cells; p=0; error bars=SD (N=3 replicates).

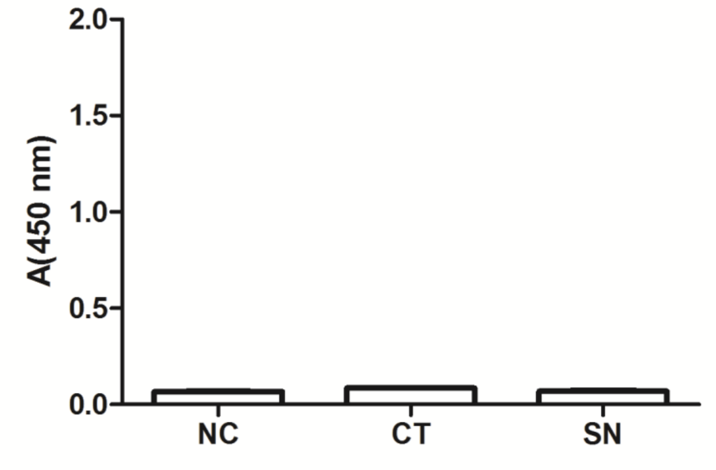

Figure S6 Expression of HER-2/neu determined by ELISA in buffer solution (NC), *MCF-7-Control* (CT) and *MCF-7-Snail* (SN) cells. ANOVA p-value = 0.1164; Error bars=SD; N=3 replicates. Y-axis scaled to demonstrate very low level of HER2/neu signal intensity (expected signal intensity in HER-2/neu positive cells is ~2 for cell lysates with the same total protein concentration [2]).

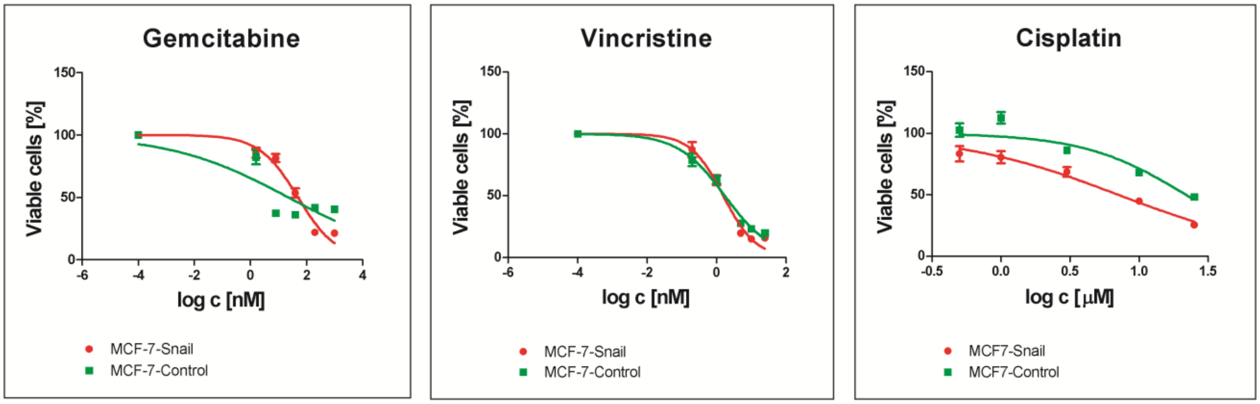

Figure S7 Examples of dose-response curves in which mesenchymal-like MCF-7-Snail cells (red) demonstrate lower sensitivity (gemcitabine), equal sensitivity (vincristine) and higher sensitivity (cisplatin) relative to epithelial-like MCF-7-Control cells (green). Curves fitted from experimental data by non-linear regression of log-transformed data using a normalized response-variable slope model (GraphPad Prism 5.01; GraphPad Software, Inc.). Error bars: SD.

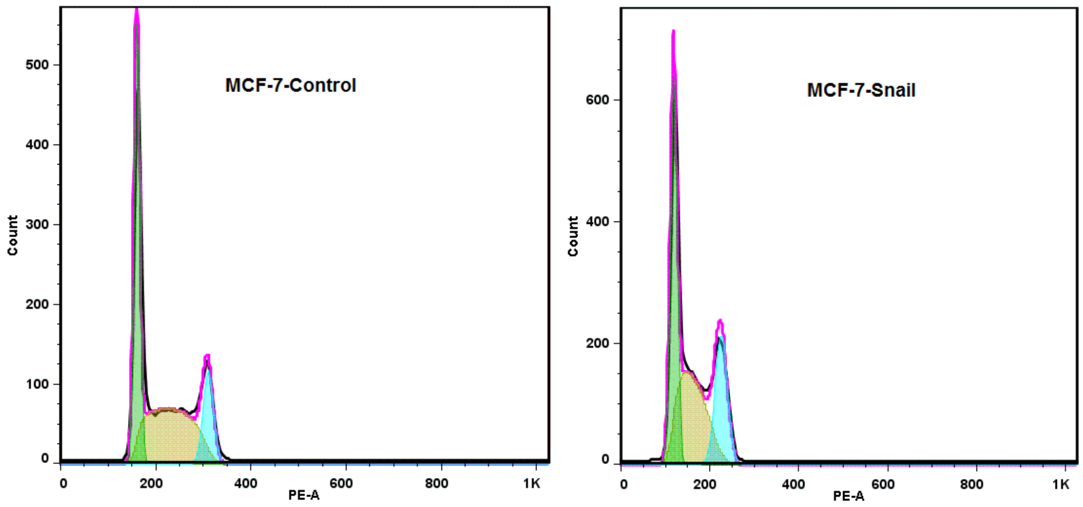

Figure S8 Cell cycle distribution analysis by DNA flow cytometry - representative examples of analysis for DNA histogram deconvolution using Dean-Jett-Fox Model. PE-A: PI-fluorescence; vertical axis: cell count. Green: G0/G1-phase; Yellow: S-phase; Magenta – G2/M-phase.

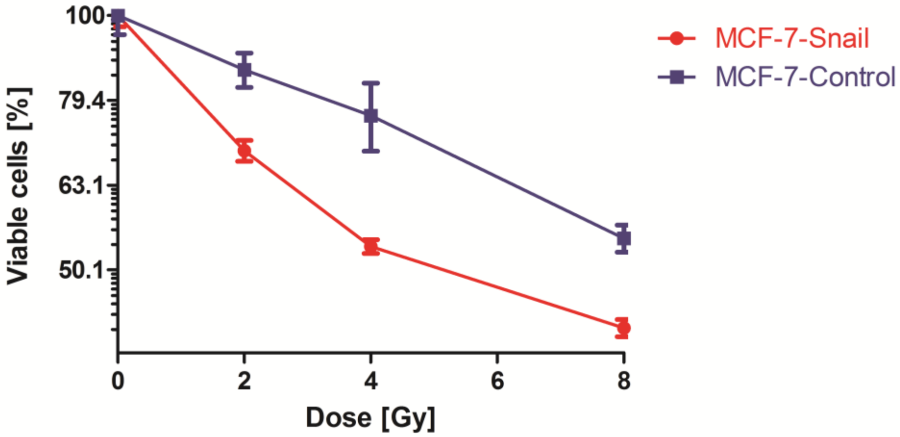

Figure S9 Radiation sensitivity curves for MCF-7-Snail and MCF-7-Control cells determined as described in Materials and Methods section and presented in semi-log plot.

Supplemental tables

Table S1 GeneGO pathway maps significantly enriched for up-regulated genes in MCF-7-Snail vs MCF-7-Control cells (FDR=0.1677)

#	Pathway Map	p-value	FDR	P/T
1	Cell cycle_Start of DNA replication in early S phase	6.576E-05	5.346E-02	17/31
2	Transport_RAN regulation pathway	3.812E-04	1.196E-01	11/18
3	DNA damage_ATM/ATR regulation of G1/S checkpoint	4.413E-04	1.196E-01	16/32
4	Ca(2+)-dependent NF-AT signaling in cardiac hypertrophy	1.013E-03	1.677E-01	17/37
5	Apoptosis and survival_Endoplasmic reticulum stress response pathway	1.154E-03	1.677E-01	21/50
6	Development_Oligodendrocyte differentiation from adult stem cells	1.237E-03	1.677E-01	20/47

Table S2 GeneGO pathway maps significantly enriched for down-regulated genes in MCF-7-Snail vs MCF-7-Control cells (FDR=0.01514)

#	Pathway Map	p-value	FDR	P/T
1	Development_Thromboxane A2 signaling pathway	1.796E-07	8.374E-05	21/36
2	Development_Ligand-independent activation of ESR1 and ESR2	2.068E-07	8.374E-05	22/39
3	Androstenedione and testosterone biosynthesis and metabolism p.2/ Rodent version	5.063E-06	8.820E-04	12/17
4	Androstenedione and testosterone biosynthesis and metabolism p.2	5.063E-06	8.820E-04	12/17
5	Development_ERBB-family signaling	5.444E-06	8.820E-04	19/36
6	Regulation of CFTR activity (normal and CF)	8.666E-06	1.056E-03	21/43
7	Immune response_IL-13 signaling via PI3K-ERK	9.126E-06	1.056E-03	18/34
8	Development_S1P3 receptor signaling pathway	1.306E-05	1.322E-03	15/26
9	Immune response_PGE2 common pathways	2.426E-05	1.879E-03	15/27
10	Apoptosis and survival_BAD phosphorylation	2.552E-05	1.879E-03	18/36
11	ENaC regulation in normal and CF airways	2.552E-05	1.879E-03	18/36
12	Ovarian cancer (main signaling cascades)	2.804E-05	1.893E-03	25/59
13	Transcription_CREB pathway	3.829E-05	2.215E-03	19/40
14	Development_Alpha-2 adrenergic receptor activation of ERK	3.829E-05	2.215E-03	19/40
15	Development_Membrane-bound ESR1: interaction with growth factors signaling	4.293E-05	2.318E-03	17/34
16	Immune response_CCR5 signaling in macrophages and T lymphocytes	5.314E-05	2.690E-03	20/44
17	Apoptosis and survival_HTR1A signaling	6.418E-05	2.888E-03	18/38

18	Development_A1 receptor signaling	6.418E-05	2.888E-03	18/38
19	Development_Hedgehog and PTH signaling pathways in bone and cartilage development	7.379E-05	3.146E-03	15/29
20	PTMs in IL-17-induced CIKS-independent signaling pathways	8.849E-05	3.446E-03	19/42
21	Ligand-independent activation of Androgen receptor in Prostate Cancer	8.934E-05	3.446E-03	24/59
22	Development_G-Proteins mediated regulation MAPK-ERK signaling	1.076E-04	3.791E-03	17/36
23	Development_A3 receptor signaling	1.076E-04	3.791E-03	17/36
24	Development_Role of IL-8 in angiogenesis	1.224E-04	3.878E-03	22/53
25	Chemotaxis_C5a-induced chemotaxis	1.245E-04	3.878E-03	14/27
26	Development_S1P1 signaling pathway	1.245E-04	3.878E-03	14/27
27	Development_TGF-beta-dependent induction of EMT via RhoA, PI3K and ILK.	1.307E-04	3.921E-03	19/43
28	Immune response_ICOS pathway in T-helper cell	1.643E-04	4.603E-03	17/37
29	Development_GM-CSF signaling	1.648E-04	4.603E-03	20/47
30	Apoptosis and survival_TNF-alpha-induced Caspase-8 signaling	1.806E-04	4.875E-03	16/34
31	Signal transduction_Activation of PKC via G-Protein coupled receptor	1.896E-04	4.875E-03	19/44
32	Cytoskeleton remodeling_Role of PKA in cytoskeleton reorganisation	1.951E-04	4.875E-03	15/31
33	Development_Thrombopoetin signaling via JAK-STAT pathway	2.055E-04	4.875E-03	12/22
34	Development_Angiotensin signaling via STATs	2.103E-04	4.875E-03	13/25
35	Development_Endothelin-1/EDNRA signaling	2.167E-04	4.875E-03	18/41
36	PGE2 pathways in cancer	2.167E-04	4.875E-03	18/41
37	IGF family signaling in colorectal cancer	2.334E-04	5.096E-03	22/55
38	Muscle contraction_Relaxin signaling pathway	2.454E-04	5.096E-03	17/38
39	G-protein signaling_Proinsulin C-peptide signaling	2.454E-04	5.096E-03	17/38
40	Mucin expression in CF airways	2.755E-04	5.580E-03	21/52
41	Development_Angiopoietin - Tie2 signaling	3.034E-04	5.995E-03	15/32
42	Influence of low doses of Arsenite on glucose uptake in adipocytes	3.466E-04	6.685E-03	11/20
43	Immune response_IL-2 activation and signaling pathway	3.802E-04	7.162E-03	19/46
44	Cytoskeleton remodeling_Keratin filaments	4.085E-04	7.353E-03	16/36
45	Immune response_IL-9 signaling pathway	4.085E-04	7.353E-03	16/36
46	Development_CNTF receptor signaling	5.102E-04	8.440E-03	14/30
47	Development_EGFR signaling pathway	5.107E-04	8.440E-03	24/65
48	Development_GDNF family signaling	5.154E-04	8.440E-03	17/40
49	Immune response_C5a signaling	5.154E-04	8.440E-03	17/40
50	Apoptosis and survival_Beta-2 adrenergic receptor anti-apoptotic action	5.210E-04	8.440E-03	9/15
51	Immune response_IFN alpha/beta signaling pathway	5.899E-04	9.065E-03	12/24
52	Main growth factor signaling cascades in multiple myeloma cells	5.946E-04	9.065E-03	16/37
53	G-protein signaling_S1P2 receptor signaling	6.039E-04	9.065E-03	11/21
54	Inhibition of neutrophil migration by proresolving lipid mediators in COPD	6.044E-04	9.065E-03	20/51
55	Development_FGFR signaling pathway	6.199E-04	9.129E-03	18/44
56	Transcription_PPAR Pathway	8.486E-04	1.179E-02	16/38
57	Immune response _IFN gamma signaling pathway	8.527E-04	1.179E-02	18/45
58	Immune response_CD28 signaling	8.527E-04	1.179E-02	18/45

59	Transport_Alpha-2 adrenergic receptor regulation of ion channels	8.589E-04	1.179E-02	13/28
60	Reproduction_GnRH signaling	9.644E-04	1.277E-02	22/60
61	Immune response_Oncostatin M signaling via MAPK in mouse cells	9.842E-04	1.277E-02	15/35
62	Cell adhesion_Gap junctions	1.002E-03	1.277E-02	11/22
63	CFTR-dependent regulation of ion channels in CF	1.002E-03	1.277E-02	11/22
64	Immune response_BCR pathway	1.009E-03	1.277E-02	17/42
65	Development_EGFR signaling via PIP3	1.027E-03	1.280E-02	10/19
66	Development_Gastrin in cell growth and proliferation	1.073E-03	1.317E-02	20/53
67	Signal transduction_Calcium signaling	1.132E-03	1.369E-02	14/32
68	Immune response_Fc epsilon RI pathway	1.157E-03	1.378E-02	18/46
69	Neurophysiological process_HTR1A receptor signaling in neuronal cells	1.289E-03	1.514E-02	13/29

Supplemental methods

Method S1 Relative expression of Snail (SNAI1) and Slug (SNAI2) genes in *MCF-7-Snail* vs. *MCF-7-Control* cells was determined by qPCR using SYBR Green method as previously described [3]. Sequences of primers are listed below:

Gene	Forward primer<br>Reverse primer	Reference
GAPDH	5'-CTCTCTGCTCCTCCTGTTCGAC-3'<br>5'-TGAGCGATGTGGCTCGGCT-3'	[4]
SNAI2	5'-GGGAGAAGCCTTTTTCTTG-3'<br>5'-TCGTCATGTTTGAGCAGGAG-3'	[5]
SNAI1	5'-CCTCCCTGTCAGATGAGGAC-3'<br>5'-CCAGGCTGAGGTATTCCTTG-3'	[6]

Method S2 Expression of ESR1 and EGFR genes in *MCF-7-Snail* vs. *MCF-7-Control* cells was determined by qPCR using TaqMan method as previously described [7] using following TaqMan Gene Expression Assays: Hs00174860_m1 (ESR1); Hs01076078_m1 (EGFR); and Hs02758991_g1 assay (GAPDH).

Method S3 The level of total HER-2/neu protein in *MCF-7-Snail* vs. *MCF-7-Control* cells was determined by ELISA using the PathScan Total HER2/ErbB2 Sandwich ELISA Kit (Cell Signaling Technology, Inc.) following the manufacturer's instructions. Cells were grown in 3 independent cell cultures to ~80% confluence and lysed using the included cell lysis buffer. Total protein concentration in cell lysates was determined with Pierce Detergent Compatible Bradford Assay Kit (Thermo Fisher Scientific) and adjusted to 80 µg/mL. Total HER-2/neu was determined by absorbance reading at 450 nm.

References

[1] Subik K, Lee JF, Baxter L, Strzepek T, Costello D, Crowley P, Xing L, Hung MC, Bonfiglio T, Hicks DG, Tang P. The Expression Patterns of ER, PR, HER2, CK5/6, EGFR, Ki-67 and AR by Immunohistochemical Analysis in Breast Cancer Cell Lines, Breast Cancer : Basic and Clinical Research 2010; 4: 35-41.

[2] PathScan® Total HER2/ErbB2 Sandwich ELISA Kit #7310; accessed 12/11/2015; http://www.cellsignal.com/products/elisa-kits/total-her2-erbb2-sandwich-elisa-kit/7310

[3] Mezencev R, Wang L, Xu W, Kim B, Sulchek TA, Daneker GW, McDonald JF. Molecular analysis of the inhibitory effect of N-acetyl-L-cysteine on the proliferation and invasiveness of pancreatic cancer cells. Anticancer Drugs. 2013;24(5):504-518

[4] Leung JC, Chan LY, Tang SC, Lam MF, Chow CW, Lim AI, Lai KN. Oxidative damages in tubular epithelial cells in IgA nephropathy: role of crosstalk between angiotensin II and aldosterone. J Transl Med. 2011 Oct 6;9:169.

[5] Louie E, Nik S, Chen JS, Schmidt M, Song B, Pacson C, Chen XF, Park S, Ju J, Chen EI. Identification of a stem-like cell population by exposing metastatic breast cancer cell lines to repetitive cycles of hypoxia and reoxygenation. Breast Cancer Res. 2010;12(6):R94.

[6] Wei P, Zhang N, Wang Y, Li D, Wang L, Sun X, Shen C, Yang Y, Zhou X, Du X.FOXM1 promotes lung adenocarcinoma invasion and metastasis by upregulating SNAIL.Int J Biol Sci. 2015;11(2):186-198.
